# A Process Similar to Autophagy Is Associated with Cytocidal Chloroquine Resistance in *Plasmodium falciparum*

**DOI:** 10.1371/journal.pone.0079059

**Published:** 2013-11-20

**Authors:** David Gaviria, Michelle F. Paguio, Lindsey B. Turnbull, Asako Tan, Amila Siriwardana, Debasish Ghosh, Michael T. Ferdig, Anthony P. Sinai, Paul D. Roepe

**Affiliations:** 1 Departments of Chemistry and of Biochemistry, Cellular and Molecular Biology, Georgetown University, Washington, District of Columbia, United States of America; 2 Eck Institute for Global Health, Department of Biology, University of Notre Dame, Notre Dame, Indiana, United States of America; 3 Department of Microbiology, Immunology and Molecular Genetics, University of Kentucky College of Medicine, Lexington, Kentucky, United States of America; Instituto de Higiene e Medicina Tropical, Portugal

## Abstract

Resistance to the cytostatic activity of the antimalarial drug chloroquine (CQ) is becoming well understood, however, resistance to cytocidal effects of CQ is largely unexplored. We find that PfCRT mutations that almost fully recapitulate *P. falciparum* cytostatic CQ resistance (CQR^CS^) as quantified by CQ IC_50_ shift, account for only 10–20% of cytocidal CQR (CQR^CC^) as quantified by CQ LD_50_ shift. Quantitative trait loci (QTL) analysis of the progeny of a chloroquine sensitive (CQS; strain HB3)×chloroquine resistant (CQR; strain Dd2) genetic cross identifies distinct genetic architectures for CQR^CS^ vs CQR^CC^ phenotypes, including identification of novel interacting chromosomal loci that influence CQ LD_50_. Candidate genes in these loci are consistent with a role for autophagy in CQR^CC^, leading us to directly examine the autophagy pathway in intraerythrocytic CQR parasites. Indirect immunofluorescence of RBC infected with synchronized CQS vs CQR trophozoite stage parasites reveals differences in the distribution of the autophagy marker protein PfATG8 coinciding with CQR^CC^. Taken together, the data show that an unusual autophagy – like process is either activated or inhibited for intraerythrocytic trophozoite parasites at LD_50_ doses (but not IC_50_ doses) of CQ, that the pathway is altered in CQR *P. falciparum*, and that it may contribute along with mutations in PfCRT to confer the CQR^CC^ phenotype.

## Introduction

Resistance to quinoline and antifolate antimalarial drugs, as well as emerging tolerance to artemisinin – based drugs [1] threatens the health of over half of the world’s population. Understanding the molecular details of *P. falciparum* and *P. vivax* antimalarial drug resistance phenomena facilitates surveillance of resistance and rapid development of more effective treatment. Two approaches for monitoring antimalarial drug resistance for *P. falciparum* malaria are available. One is analysis of clinical data to assess efficacy of specific treatments, the second is *in vitro* analysis of parasite strains or patient isolates to quantify their susceptibility to specific drugs. With the discovery of key genetic mutations in *P. falciparum* that confer resistance to either antifolate [2], [3] or quinoline – based [4] antimalarial drugs, rapid field-based surveillance of the geographic spread of existing drug resistant malaria is now possible, as is personalized delivery of second tier drug therapy to patients infected with a specific drug resistant strain.

Many antimicrobial drugs are both cytostatic and cytocidal, including quinoline antimalarial drugs such as CQ [5]–[7]. That is, under certain conditions a drug slows the rate of cell growth or impairs cell division such that the rate of proliferation of a mass population of the microbe is reduced, and under other conditions the drug kills the microbial cell. Often, cytocidal (cell kill) activity requires higher dose of drug, longer drug exposure time, or both. Cytostatic potency is usually quantified via IC_50_ values (the dose of drug at which growth is inhibited by 50% relative to control), whereas cytocidal potency is quantified via LD_50_ values (the dose of drug that kills 50% of a microbial population).

However, to date, all laboratory based quantification of antimalarial drug potency, and hence quantification of all antimalarial drug resistance phenomena, has been done with IC_50_ values alone. Again, IC_50_ assays quantify the concentration of drug required to inhibit proliferation of parasite populations by 50%. Drug IC_50_ for *P. falciparum* are typically measured in red blood cell culture suspensions in the continuous presence of serially diluted concentrations of the drug. Such quantification has proved critical for defining the genetics and biochemistry behind resistance to the cytostatic effects of CQ (CQR^CS^) [4], [8]–[11] and for identifying new drug leads with excellent cytostatic potential vs CQR malaria [12]. It is sometimes assumed that IC_50_ values measure the “cell kill” effect of a drug. Although this can be true in specific cases, in many others this is not the case. Use of IC_50_ values alone can over-estimate the cytocidal activity of a drug and can under-estimate potential parasite survival in the presence of higher (clinically relevant) levels of the drug. Since laboratory CQ IC_50_ are in the 10^−9^–10^−8 ^M range, but peak plasma levels of CQ in patients are ∼1000 times higher (10^−6^–10^−5 ^M; see [13]–[15]), clarification of these points is essential for fully understanding CQR.

That is, although the mechanism of CQR^CS^ is becoming clear, much less is known about resistance to the cytocidal effects of CQ (CQR^CC^) (or cytocidal resistance vs any other antimalarial drug for that matter). This is a critical piece of missing information, given that parasite *survival* determines the rate of adaptation to selection by drugs. Only recently has it been possible to efficiently and reproducibly quantify LD_50_ for some antimalarial drugs, and rapid quantification of LD_50_ differences for drug sensitive vs drug resistant parasites are found in only one paper to our knowledge [6]. Formally, without additional information the ratio of drug IC_50_ values for drug sensitive vs drug resistant parasites expresses the degree of cytostatic resistance, whereas LD_50_ ratios express the degree of cytocidal resistance [6].

For any drug (anti-tumor, anti-bacterial, anti-fungal, anti-parasitic) it is theoretically possible that the molecular mechanisms controlling cytostatic and cytocidal activities could overlap. If this is the case, then the molecular mechanisms of resistance to those two distinct layers of drug pharmacology would also presumably overlap. In other cases, particularly when significantly different concentrations of drug are needed to kill the relevant cell vs merely slow its growth, molecular targets for the two layers of drug activity may differ, and then the mechanisms of cytostatic vs cytocidal resistance could be distinct. This has been observed in some examples of multidrug resistance, for example, overexpression of P- glycoprotein and other plasma membrane events can be particularly relevant for antitumor drug cytostatic resistance, whereas altered induction or regulation of apoptosis (programmed cell death) is particularly important in antitumor drug cytocidal resistance [16].

Perhaps relatedly, we recently reported that although decreased CQ accumulation within CQR *P. falciparum* is clearly related to elevated CQ IC_50_, it is not necessarily relevant for elevated CQ LD_50_ [17]. Surprisingly, much higher concentrations of CQ or fluorescent NBD - CQ can be found within parasites exhibiting cytocidal CQ resistance, even though reduced drug uptake is generally accepted to be the principle basis of CQR [17]. We have also recently found that, for both 4 amino quinolines similar to CQ and quinoline methanols similar to quinine (QN), IC_50_ is correlated with the ability of the drugs to inhibit hemozoin crystallization under close to physiologic conditions, but LD_50_ for the same drugs is not [18], [19]. Patterns of IC_50_ vs LD_50_ for a variety of quinoline drugs also suggest that the mechanisms for cytostatic vs cytocidal CQ resistance in *P. falciparum* are not entirely the same [6]. Taken together these data suggest that the cellular targets relevant for quinoline antimalarial drug cytocidal activities may differ from targets for cytostatic activities. Drug DV localization and drug/heme binding is the likely basis of CQ cytostatic pharmacology, but perhaps not the entire basis of CQ cytocidal pharmacology [17]–[19]. Since drug resistance is due to disruption of drug/drug target interactions, then different targets for cytostatic vs cytocidal effects predict distinct mechanisms of cytostatic vs cytocidal resistance, unless resistance is merely due to increased catabolism of the drug (which is not the case for CQR or QNR in *P. falciparum*).

With respect to *P. falciparum* CQR^CS^, elevated CQ IC_50_ and reduced parasite CQ accumulation are well correlated with mutations in the DV membrane CQ transporter PfCRT [11], suggesting that CQR^CS^ is due to decreased drug accessibility to heme targets within the parasite DV [20]. However, resistance to the cytocidal effects of CQ is predicted to include alterations in additional targets, access to these additional targets [17], and/or to encompass mutations in key regulators of *P. falciparum* cell death pathways as recently hypothesized [21]. With regard to this last point, being a single celled organism, and due to the lack of caspase genes and other genes that encode key apoptosis regulators within the *P. falciparum* genome, it is questionable whether the canonical apoptosis pathway is the cause of drug - induced cell death for the malarial parasite [21]. Some evidence for an apoptotic - like cell death pathway for *P. falciparum* involving metacaspases has been presented [22]–[25] but there is disagreement on how relevant these observations are for *P. falciparum* death via different drugs [26]. More importantly, no molecular alterations in apoptosis have been found for CQR malaria.

These points led us to rank the progeny of the HB3 (CQS) × Dd2 (CQR) *P. falciparum* cross for CQ LD_50_ and to perform LD_50_– directed QTL analysis. Progeny of this genetic cross have proven invaluable to analysis of CQR phenomena [4], [8], [10], [27]. By quantifying CQ IC_50_ values for these progeny, a single locus on chr7 was previously identified as controlling the difference between CQR and CQS strain CQ IC_50_ [4]. Subsequent sequencing, *in vitro* drug pressure, and transfection results showed that multiple amino acid substitution mutations within a single gene in the chr7 locus, *pfcrt,* causes the large shift in IC_50_ values that has historically defined CQR and CQS status [4], [28], [29]. Allelic exchange experiments that directly replaced the “wild type” CQS associated *pfcrt* allele with mutant CQR associated *pfcrt* resulted in elevated CQ IC_50_ without the need to condition or select cells with CQ [30]. The degree to which CQ IC_50_ was elevated for these allelic exchange transfectants was very similar to that seen for highly drug selected CQR strains (70%–90% of the corresponding strain IC_50_ shift, see [30]), suggesting that the presence of mutant PfCRT protein was in-and-of-itself sufficient (or nearly sufficient) for conversion to a CQR phenotype. However, subsequent QTL analyses suggested that additional genetic components, such as inheritance of different chr5 loci containing mutations and varying copies of *pfmdr1*, may combine with PfCRT mutations in various isolates to confer the range of CQ IC_50_ and variable IC_50_ patterns for different drugs now known to exist across the globe [8]–[10], [27]. In contrast, QTL analysis in this paper shows a complete lack of the key chr5 locus previously identified for CQR^CS^, and identifies additional and unique genomic loci specific to CQR^CC^. Examination of genes in these loci suggests candidate pathways that may contribute to CQR^CC^. Relatedly, LD_50_ analysis of *pfcrt* allelic exchange progeny further supports our overall conclusion that although some PfCRT mutations in and of themselves confer nearly complete resistance to CQ cytostatic effects as defined by IC_50_ shift (see [30]), they are less important for cytocidal CQ resistance (CQR^CC^) as defined by LD_50_ shift. Using antibodies to the autophagy indicator protein ATG8 and high - resolution fluorescence microscopy, we find that a drug-induced autophagy-like cascade is dysregulated in CQR^CC^ *P. falciparum*. Taken together, and considered alongside additional recent work with the related parasite *T. gondii* [31], our data suggest that a dysregulated autophagy-like process, combined with PfCRT mutations, promotes elevated CQ LD_50_ in CQR *P. falciparum*.

## Materials and Methods

### Cell Culture

*P. falciparum* strains HB3, Dd2, and their genetic cross progeny were acquired from the Malaria Research and Reference Reagent Resource Center (Manassas, VA), while GC03 transfectants (see [30]) were a kind gift from DA Fidock (Columbia University, New York, NY). All cultures were grown in complete media at 37°C as described [6] using type O^+^ human blood and human serum. Parasites were highly synchronized 3 times prior to experiments at 0, 10, and 52 hours using a solution of 5% sorbitol as described [7]. Parasites were incubated at 37°C/5%CO_2_ in either complete, starvation, or CQ containing media for 6 hours. When quantification of PfATG8 puncta was desired, CQ pulse was done for 6 hours using highly synchronized parasites at the mid trophozoite stage, followed by washing to remove drug as described [6].

### IC_50_ and LD_50_ Quantification

Our assay for IC_50_ has been published previously [32] and has been used extensively and validated by many other laboratories. Our semi-high-throughput assay for LD_50_ quantification has recently been published elsewhere [6]. In brief, for LD_50_ quantification asynchronous *P. falciparum* cultures at 2% hematocrit and 2% parasitemia were treated with CQ in bolus fashion for 6 hr and then the drug was completely washed away [6]. After 48 hr of further incubation at 37°C under 5% CO_2_, Sybr Green I was added and fluorescence was measured at excitation and emission wavelengths of 485 nm and 538 nm, respectively [6]. For IC_50_ quantification initial parasitemia is reduced to 0.5% and cells remain in the constant presence of lower levels of drug [32]. In previous work [6] we defined differences in LD_50_ for synchronized vs asynchronous culture and vs other variables. LD_50_ and IC_50_ data for individual assays (each assay performed with triplicate plating of parasites treated at a given dose) were fit to a sigmoidal function using SigmaPlot 9.0 (San Jose, CA), and IC_50_ or LD_50_ values were calculated from at least three individual assays (at least 9 determinations in total) and averaged. Outgrowth in the LD_50_ assay 48 hr after drug is washed away represents expansion of live cells that survived cytocidal doseages of drug, as supported by staining for outgrowth after different incubation times (see [6] for detailed discussion of this point and related issues). CQ IC_50_ and LD_50_ values for laboratory strains analyzed in this paper are found in Table 1, and CQ LD_50_ values for HB3×Dd2 cross progeny can be found in Table S1.

**Table 1:** Average CQ IC_50_ and CQ LD_50_ (+/− [S.E.M.]; at least 3 separate assays in each case, each assay done in triplicate for ≥9 determinations each) for strains GC03 (CQS), Dd2 (CQR), 7G8 (CQR), C2GC03 (control CQS transfectant), C4Dd2 (CQS strain GC03 after allelic exchange with CQR Dd2 *pfcrt* allele), and C67G8 (CQS strain GC03 after allelic exchange with CQR 7G8 *pfcrt* allele).

Strain	CQ IC50 (nM +/− [SEM])	CQ LD50 (µM +/− [SEM])
GC03	22.1 [1.7]	.0531 [0.005]
Dd2	206 [26.3]	15.7 [1.82]
7G8	161 [14.1]	3.99 [0.62]
C2GC03	24.4 [1.9]	.096 [.008]
C4Dd2	187 [21.0] (90%)	1.10 [0.082] (7%)
C67G8	128 [12.8] (79%)	0.91 [0.067] (23%)

Also shown for C4Dd2 and C67G8 are (% IC50 or % LD50, relative to Dd2 or 7G8 values, respectively). See [30] for description of the allelic exchange transfectants. Note IC_50_ assays are done in the continuous presence of low dose CQ for >48 hrs [33] whereas LD_50_ assays require higher doses of CQ given as a 6 hr bolus [6].

### QTL Analysis

Using mean LD_50_ for each of the progeny of the HB3×Dd2 cross, genome-wide scans were run using Pseudomarker 2.04 to detect quantitative trait loci (QTL) associated with the drug response. Genome-wide significance thresholds which correct for multiple testing errors were determined by permutation testing (n = 1000 permutations). The strength of the association between a given locus and the trait (LD_50_) is expressed as a logarithm of odds (LOD) score. Loci that exceeded the 99^th^ percentile (p<0.01), the 95^th^ percentile (p<0.05), and the 37^th^ percentile (p<0.63) were identified respectively as highly significant, significant, and suggestive QTL. Two-dimensional linear regression genome scans were run to test for potential loci interactions and joint LOD scores were calculated to identify significance interactions.

Candidate genes within QTL loci were selected as described [33]. In brief, candidate genes were selected based on four selection criteria: (1) genomic position, (2) structural polymorphisms, (3) correlation between LD_50_ and expression phenotypes for each parasite, and (4) gene annotations and enrichment analysis. PlasmoDB version 9.3 was used for SNP density scoring (CDS), and gene annotations. Gene enrichment analysis for biological processes and molecular functions was performed using DAVID [34]. Expression phenotypes for genes within our loci were taken from [35]. Permutation testing (n = 1000) confirmed significance of GO enriched terms within the loci of interest.

### Preparation of Affinity Purified ATG8 IgG and ATG8 Monoclonal Antibody 2K19

A RACE validated cDNA encoding *Toxoplasma gondii* TgATG8 (Toxo.DB accession number TGGT1_003400) was used as template to amplify the full length coding region using the primers (CACCATGCCATCGATTCGCGACGAAGTGTCC and TTACCCCAGAGTGTTCTCTGAAGAGTATTCCA CGTACA). The amplicon was subcloned into pET100 establishing an in frame N-terminal hexa-His tag. Following expression in E. coli, the recombinant His-TgATG8 was purified on Ni-NTA magnetic beads (Invitrogen) and used as the immunogen to immunize a single rabbit (Cocalico, Reamstown, PA). The resulting antiserum was affinity purified using the bead immobilized crosslinked antigen and eluted using low pH.

Generation of a monoclonal antibody was contracted to AbMART.com (Shanghai, China) and accomplished using a synthetic polyprotein containing 6 tandemly arrayed epitopes. Epitopes were selected on the basis of the highest homology between TgATG8 and PfATG8. Supernatants from clones yielding >3 fold signal in an ELISA at 1∶128K dilution were screened by IFA on both *Toxoplasma* and *Plasmodium*. Multiple clones recognizing the epitope HRIRAKYPNR in *Toxoplasma* (HKIRSKYPNR in *Plasmodium*) gave excellent reactivity in both organisms. The clone 2K19 was selected for detailed work as it was found to be an IgG1 isotype (data not shown) and gave the best defined signal in both organisms.

### Immunohistochemistry

For starvation treatments, cells at the mid trophozoite stage were pelleted and resuspended in HBS supplemented with 0.1 mg mL^−1^ hypoxanthine, 25 mM HEPES (pH 7.3), and 20 mM sodium bicarbonate. Cells were gassed and incubated at 37°C for a desired interval (typically 6 hours) before fixation. For CQ treatments, highly synchronized mid stage trophozoites were treated as described [6], [7] using drug concentrations noted in the text. Resultant cell pellets were resuspended in complete media and treated as below.

Cells were washed 3 times with 25 mM HEPES pH 7.3, fixed with 4% formaldehyde/0.0075% glutaraldehyde in PBS for 30 minutes, permeabilized with 0.1% Triton X-100 for 10 minutes, reduced with 0.3 mg mL^−1^ sodium triacetoxyborohydride for 10 minutes, blocked with 5% goat serum for 1 hour, and sequentially treated with antibodies (1∶500) diluted in 5% goat serum/PBS Tween-20 with PBS washes in between; antibody treatments lasted 1 hour at 37°C in the dark. For experiments involving mouse monoclonal 2K19, the primary antibody solution was prepared at 1∶500 and the secondary (typically goat anti mouse DyLight488) at 1∶500. For experiments involving antisera or purified IgG from rat or rabbit, primary solutions were prepared at 1∶500 and secondary solutions (goat anti rat AlexaFluor594 and goat anti rabbit DyLight488 or DyLight649) at 1∶500. Cells were attached to #1.5 coverslips and mounted using “Fluorogel” mounting media. Samples were imaged using a customized Perkin – Elmer spinning disk confocal microscope with 405 and 491 nm laser lines, typically at 200 ms exposure and 35% laser power [36].

### Cell Fluorescence Data Analysis

Images were iteratively deconvolved using a point spread function obtained under identical imaging conditions (via doping one sample with fluorescent beads) and running multiple iterations in AutoQuantX2 [36]. Images were further processed and overlayed using Imaris 7.4.2 software. Using the “spots” routine in Imaris 7.4.2, puncta were defined and distances were measured from each spot to a single point within the DV as defined by the center of hemozoin optical density (see [36] and Scheme S1). These distances were exported to Excel and the resulting data were plotted as number of puncta vs distance from hemozoin.

### Western Blot

Western blots were done as previously described [7] with slight modification. Fractionation of synchronized iRBC was as described [7]. SDS-PAGE gels (15% acrylamide) were pre loaded with lysed trophozoite-infected RBCs, electrophoresed and transferred to nitrocellulose over night at 4°C. Blots were blocked with 10% dry milk (Biorad)/PBS, washed×3 with PBS/0.1% Tween-20, labeled with rabbit anti-ATG8 antiserum (1∶10,000), washed again, and incubated with anti-rabbit HRP secondary antibody (1∶5000).

## Results

Using a more rapid SybrGreen assay [32] in place of traditional ^3^H-hypoxanthine incorporation, our quantification of CQ IC_50_ for *pfcrt* transfectants agrees with that published previously (Table 1). Clones C4^Dd2^ and C6^7G8^ [30] show approximately 8– fold and 6– fold shifted CQ IC_50_, relative to control transfectants (C2^GC03^) or the parental CQS strain GCO3 (Table 1; e.g. 187 nM/24 nM, C4^Dd2^ vs C2^GCO3^). That is, cytostatic CQ resistance for these clones is very close to that seen for laboratory strains Dd2 and 7G8 as noted earlier [30], suggesting that the Dd2 mutant PfCRT isoform and the 7G8 mutant PfCRT isoform are necessary and sufficient (or nearly sufficient) for the IC_50_ quantified CQR phenotypes in these widely studied drug selected strains. As hypothesized previously [30], it is perhaps possible that somewhat lower expression of PfCRT in the allelic exchange transfectants relative to the laboratory strains might be responsible for the measured IC_50_ shifts of 80% –90% of that observed in strains Dd2 and 7G8 (Table 1 and see also [37]).

Regardless, recently we showed that when expressed as a ratio of LD_50_ (dose required to kill 50% of parasites), “cytocidal” CQR was not the same as “cytostatic” CQR defined by IC_50_ ratios [6]. When CQ LD_50_ are quantified for CQR clones C4^Dd2^ and C6^7G8^ as described [6] and the ratio vs control clone C2^GCO3^ calculated, we find that these clones are 10– fold and 8– fold cytocidal CQR (“CQR^CC^”), whereas strains Dd2 and 7G8 are 130– fold and 35– fold CQR^CC^, respectively (Table 1 and see also [6]). That is, in contrast to exhibiting 80%–90% of the CQ IC_50_ shifts, the allelic exchange transfectants exhibit only 7%–23% of the CQ LD_50_ shifts for laboratory strains harboring the same PfCRT mutant isoform (Table 1). This indicates that although mutant PfCRT protein is indeed responsible for most of the CQR^CS^ phenotype (measured by CQ IC_50_), it makes a smaller contribution to the CQR^CC^ phenotype (measured by CQ LD_50_) observed in the same strains.

Thus, we leveraged a QTL mapping approach within an available genetic cross to compare genetic profiles of the two phenotypes and to search for additional genetic loci contributing to CQR^CC^. We quantified CQ LD_50_ for progeny of the HB3 (CQS) × Dd2 (CQR) cross using our recently described high throughput assay ([6]; see Table S1 for CQ LD_50_ values). We were able to identify three major QTL loci associated with LD_50_. First, similar to CQ IC_50_– directed QTL [4] the chr7 locus harboring *pfcrt* segregates with the elevated LD_50_ phenotype (not shown, see [4]). The LOD score (∼21) is lower than the LOD score when IC_50_ is the measured phenotype (∼40; see [4], [10]). We emphasize that the magnitude of the LOD score reveals how well a locus segregates with a phenotype across a given population (in this case, progeny from the HB3 × Dd2 cross), but does not define the relative biochemical contribution of proteins encoded by that locus to the phenotype in question. Behavior of *pfcrt* transfectants that isolate PfCRT mutations in a common genetic background shows that the biochemical contribution of mutant PfCRT to elevated CQ LD_50_ is lower relative to its contribution to elevated CQ IC_50_ (Table 1), nonetheless, PfCRT mutations remain a prominent contribution to the mechanism of elevated LD_50_.

**Figure 1:**
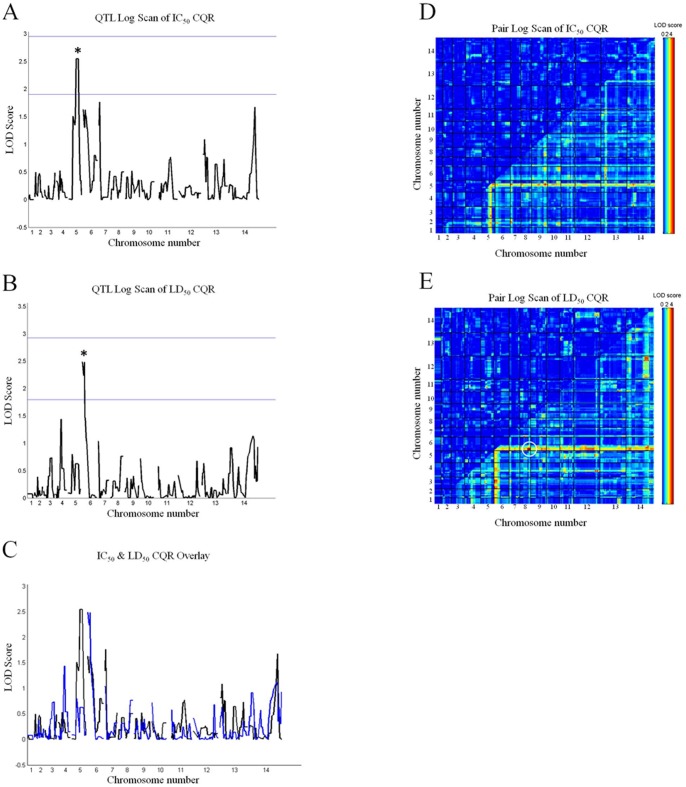
LD_50_ vs IC_50_ directed QTL analyses for CQR HB3 × Dd2 cross progeny. A) IC_50_ QTL scan for CQR progeny shows a peak on chr5 (asterix) that encompasses *pfmdr1* as previously described [10]. Notably, the chr6 locus that is pertinent for the LD_50_ scan (see Fig. 1B) does not pass the suggestive threshold on this IC_50_ scan. B) LD_50_ QTL scan for CQR progeny shows a peak on chr6 (asterix; L.O.D. = 2.5, passing the suggestive threshold). The locus that includes *pfmdr1* does not pass the suggestive threshold for this scan (see also Fig. 1C). C) To more clearly highlight the differences in genetic architecture for LD_50_ vs IC_50_ phenotypes, an overlay of the two QTL scans is shown. The CQR progeny LD_50_ QTL scan is shown in blue, while the CQR progeny IC_50_ QTL scan is in black. The overlay shows quite clearly that the *pfmdr1* locus does not factor at all into the LD_50_ phenotype, and that the chr6 locus does not factor at all into the IC_50_ phenotype. Thus the IC_50_ and LD_50_ phenotypes are genetically distinct. D) Similarly, the interaction locus between chr6 & 8 (see text) does not appear on a IC_50_ pair-wise scan. However, additive effects between chr5 and chr7 loci are seen, as previously reported [10]. E) Pair-wise scan of the CQR progeny shows that chr6 and chr8 loci (circle) have additive effects on LD_50_ (L.O.D. = 4.3).

Second, two new chromosomal loci, not previously associated with any CQR phenomena, are associated with elevated CQ LD_50_ in CQR progeny of the HB3 × Dd2 cross (Fig. 1). One is a novel contribution from chr6 (0 cM –17.3 cM) that can increase CQ LD_50_ for CQR progeny of the cross (Fig. 1B). Furthermore, this locus interacts with chr8 (77.5 cM) with significant additive effects (Fig. 1E). In addition to defining a genetic architecture for CQ LD_50_ that is distinct from that previously defined for CQ IC_50_ (Fig. 1C), the loci contain genes that may contribute to CQR^CC^ but that are not involved in CQR^CS^. In contrast, QTL scans of IC_50_ for the same CQR progeny identify a segment of chr5 (carrying the *pfmdr1* amplicon) as contributing to elevated IC_50_ (Fig. 1A, [10]), and as described previously [10], pairwise interaction between the chr5 segment and the chr7 locus is associated with elevated IC_50_ in the CQR progeny (Fig. 1D). Results from many studies show that the relevant gene in this segment of chr5 is likely *pfmdr1* [38]–[41]. However, importantly, LD_50_ - directed QTL analysis does not identify this chr5 fragment (Fig. 1C) or the chr5×chr7 pairwise interaction. Instead, it isolates a fragment of chr6 (Fig. 1B), and defines a chr6 × chr8 pairwise interaction relevant for elevated LD_50_ (Fig. 1E). In sum, these data force us to conclude that the LD_50_ and IC_50_ phenotypes share a critical feature (mutant PfCRT found on chr7) but are otherwise genetically distinct.

Our interpretation is that the CQR^CC^ and CQR^CS^ mechanisms overlap (each requires mutant PfCRT), but that the relative contribution of PfCRT to CQR^CC^ is less than, or mechanistically distinct from, its contribution to CQR^CS^ (Table 1) and that high level CQR^CC^ requires additional factors (Fig. 1B, 1E). The question then becomes how might genes in chr7, chr6, and chr8 loci act together to elevate CQ LD_50_ up to 250 fold (Table S1; note CQ LD_50_ = 32 µM for 3BA6), when Dd2 mutant PfCRT in and of itself only appears to elevate CQ LD_50_ 10 fold (Table 1)?

Inspection of the chr6 and chr8 loci reveals multiple genes involved in processes linked to “vesicle traffic”, “proteasome function/proteolysis” and “lipid metabolism” (Table S2 & S3). For example, at least 6 genes within the chr8 locus encode proteins putatively involved in the proteasome pathway (Table S3). Candidate genes were also ranked based on the sequence similarity of strain HB3 (CQS) and strain Dd2 (CQR), and the correlation of expression levels with the LD_50_ phenotype (see Tables S2 & S3). Thus we used four previously vetted methods [33] to identify genes or pathways that are most likely relevant to LD_50_. Interesting genes within the chr6 and chr8 loci that have high CDS scores include putative orthologues of autophagy genes ATG11 and ATG14, several key metabolic regulators, multiple kinases, and a putative E3 ubiquitin ligase (Table S2 & S3). Also of note are several genes in the chr6 locus linked to response to oxidative stress. Due to the nature of chromosomal fragment inheritances in the HB3 × Dd2 cross, the chr6 × chr8 additive effect defines a much smaller region of the chr6 segment. This segment harbors only 20 genes, with 7 of those encoding proteins involved in lipid metabolism (see Discussion).

Elevated LD_50_ indicates resistance to cell death. Cell death is often mediated by signal transduction that controls a programmed cell death (PCD) pathway [21]. Importantly then, we find that the chr6 and chr8 loci do not harbor any candidate Pf metacaspases [23] or other molecules that typically regulate apoptotic PCD. That is, we find no genetic evidence from the HB3 × Dd2 cross for atypical apoptosis related to CQR^CC^. Autophagy (“self eating” upon starvation or stress) is an alternate pathway that has been linked to cell death for several cell types [42]–[44], including the related apicomplexan parasite *T. gondii* [31], [45]. It is an orchestrated, vesicle mediated, proteolysis/degradative pathway that is distinct from apoptosis. Since apoptosis genes were not found in chr6 or chr8 loci, and since “vesicle traffic”, “proteasome/proteolysis”, “lipid metabolism”, “oxidative stress” and autophagy pathways often overlap mechanistically (see Discussion), we wondered if altered autophagy might be related to LD_50_.

**Figure 2:**
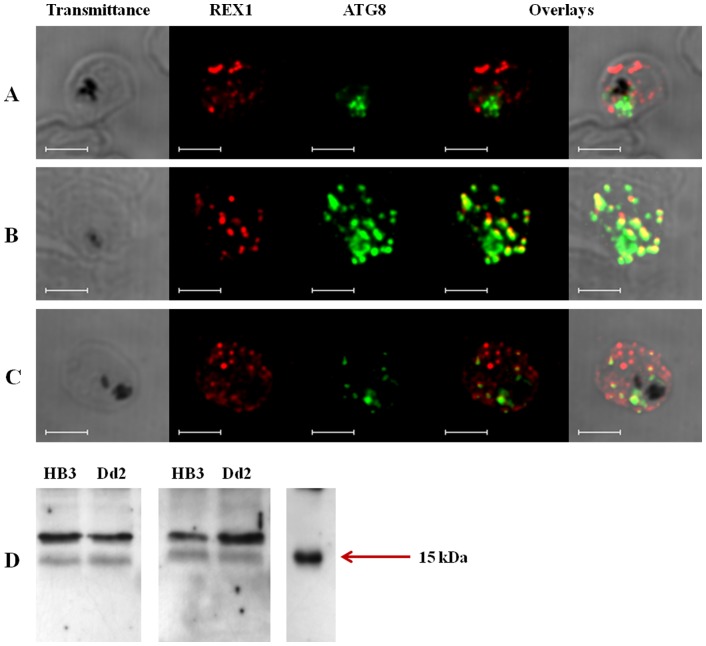
PfATG8– positive puncta. Shown are puncta for (A) control HB3 iRBC grown under normal culture conditions (B) HB3 iRBC grown for 6 hours under starvation conditions (see Methods) and (C) HB3 iRBC grown under starvation conditions plus the autophagy inhibitor 3 methyl adenine (3 MA). Shown are transmittance (left), immunofluorescence vs antiPfREX1 (Maurer’s cleft marker; red; second column), immunofluorescence vs antiTgATG8 (cross reacts with PfATG8; green, third column) and overlays (right). Bar = 5 µm. Also shown (D) are western blot data for iRBC harboring HB3 (CQS) and Dd2 (CQR) trophozoites grown under control culture conditions. Two separate gels for two independent sets of samples (two iRBC isolations for each culture) are shown. We note our data show a clear doublet at 15 and 17 kDa, similar to all other studies of eukaryotic ATG8 protein of which we are aware except one [47], which resolves only a single band instead of the usual doublet with a polyclonal antisera raised against a recombinant GST-PfATG8 fusion. We suggest three possible reasons for the discrepancy: 1) we use higher density [15% acrylate] gels relative to [47] in order to resolve the low mass doublet, 2) we do not solubilize parasites with saponin as in [47] which would release de - lipidated ATG8 into wash supernatant, 3) perhaps abundance of one PfATG8 species (presumably de lipidated) is higher in trophozoites relative to schizonts examined in [47].

**Figure 3:**
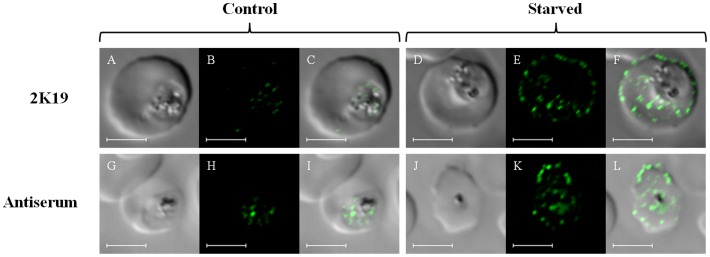
Comparison of antisera vs monoclonal Ab staining. Shown are results for TgATG8 antisera (bottom row) vs staining using a monoclonal antibody raised vs a highly conserved Apicomplexan ATG8 epitope (top row, see methods). Panels A,G,D,J are transmittance, B,H,E,K are ATG8 fluorescence, and C,I,F,L are overlay, respectively. Left side is control intraerythrocytic HB3 *P. falciparum*; right side is HB3 starved for 6 hrs as described in methods. Bar = 5 µm.

However, no experiments to our knowledge have been done to test if autophagy occurs in intraerythrocytic *P. falciparum*. The universal stimulus for autophagy is starvation, which also unambiguously induces cell death. An unambiguous feature of induction of autophagy in eukaryotes is redistribution of ATG8 protein (called LcIII in mammals) from a more localized and diffuse pattern to a more widely disbursed, more punctate pattern that defines the sites of autophagosome formation and/or recruitment of autophagosomal “cargo” [46]. Specific ATG8 antisera raised vs. *T. gondii* ATG8 protein (anti TgATG8) show excellent cross reactivity vs *P. falciparum* ATG8 because PfATG8 is ∼70% identical to TgATG8 [21]. We easily identify a PfATG8 doublet at predicted masses of 15 & 17 kDa in immunoblots of fractionated parasites (Fig. 2D) that is consistent with well known de-lipidated and lipidated forms of ATG8 [46]. IFA analysis using ATG8 antiserum suggests an autophagy-like process is active in intraerythrocytic *P. falciparum* trophozoites (Fig. 2,3). As expected, control iRBC trophozoite parasites grown in complete media show a cytosolic PfATG8 distribution (Fig. 2A, green) that appears somewhat punctate, perhaps due to at least partial localization to the apicoplast (see below and [47]). However, notably, when highly synchronized trophozoites are placed in starvation medium for 6 hrs, PfATG8 is redistributed in a much more expanded punctate fashion (Fig. 2B, green), as recently reported [21]. Closer inspection reveals puncta at the parasite periphery, possibly near the RBC membrane. Co – staining with a marker for Maurer’s cleft (anti - PfREX1, red) shows that some PfATG8 appears to be routed to very near Maurer’s cleft (MC) (yellow dots, Fig. 2B middle). The well - characterized inhibitor of autophagy, 3-methyl adenine (3-MA) partially reverses the starvation induced PfATG8 puncta redistribution (Fig. 2C), similar to what has recently been found for *T. gondii* [31]. Affinity purified IgG from the antisera as well as monoclonal antibody 2K19 raised against a highly conserved Apicomplexan ATG8 motif (see methods) yield results similar to polyclonal TgATG8 antisera (Fig. 3). Another recent report [47] presents data consistent with some localization of PfATG8 to the parasite apicoplast for control schizonts growing in normal media. We also obtain data consistent with partial (but not exclusive) localization of PfATG8 to the apicoplast for control late trophozoites/early schizonts, by co – staining for apicoplast – specific PfACP protein (see Fig. S1). We note that the trophozoite (feeding) and schizont (nuclear division/parasite replication) stages of parasite development would be expected to utilize autophagy machinery in different ways [48], [49] and that further study of PfATG8 in trophozoites vs schizonts is warranted.

**Figure 4:**
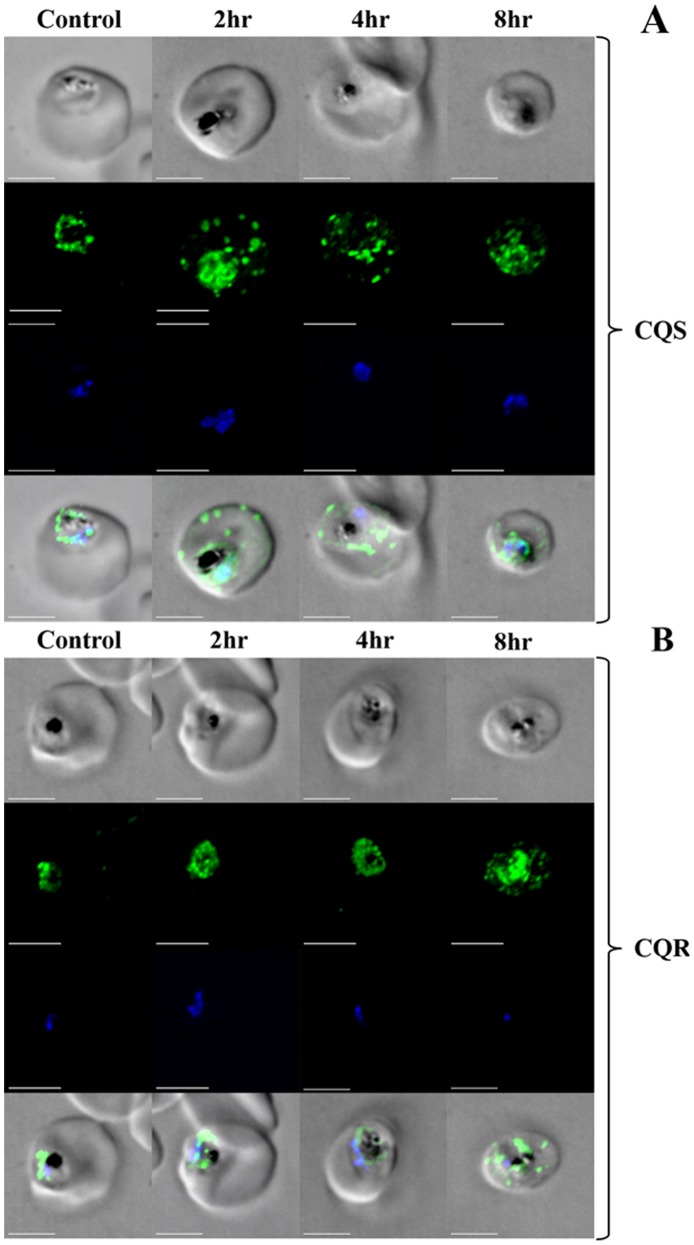
PfATG8 (green) re distribution, CQ vs time. CQS (strain HB3; top panel A) and CQR (strain Dd2; bottom panel B) parasites were grown in control media (left column) or in media plus 2×LD_50_ dose of CQ (250 nM for HB3 Fig. 4A, 30 µM for Dd2, Fig. 4B) for 2, 4 or 8 hrs (column 2,3,4 respectively). The top row in panels A and B is transmittance, the second (green) is staining for PfATG8, the third is DAPI to visualize the single trophozoite nucleus, and the bottom row for each panel presents the overlay. Fluorescence acquired at 35% power, 200 ms, 642 nm; emission 700/75 nm dichroic, 690 nm cut-off. Bar = 5 µm.

We next tested if cytocidal levels of CQ induced similar PfATG8 redistribution. Indeed, CQS parasites show similar extensively distributed PfATG8 puncta when they are treated at 2×LD_50_ cytocidal dose of CQ (Fig. 4A), but not when they are treated with 2×IC_50_ cytostatic dose (Fig. S2). Thus the autophagy – like cascade induced by starvation is also involved in the response to cytocidal levels of CQ, but not response to cytostatic levels. When CQR parasites are treated with the same absolute dose (2×CQS LD_50_ dose; 250 nM), we do not observe the punctate redistribution of PfATG8 (see below). However, when CQR parasites are treated with 2×CQR parasite LD_50_ dose (*e.g.* a similar effective pharmacologic dose, ∼32 µM for strain Dd2, see [6]) some PfATG8 redistribution is observed (Fig. 4B), but the ATG8 response appears somewhat muted.

**Figure 5:**
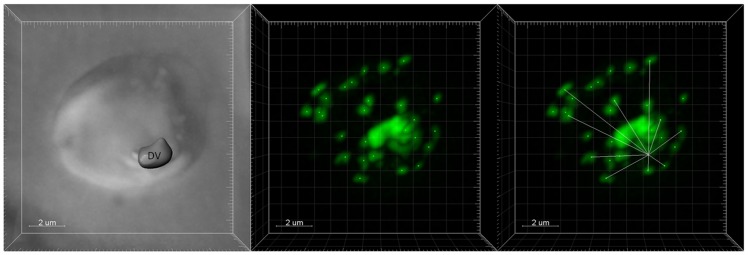
Semi automated computational method for quantifying the distribution of ATG8 puncta relative to hemozoin crystals within the DV. (A) Hemozoin within the DV is detected by transmittance, the outline of <10% transmittance is defined (labeled “DV”, left panel) and the center of this outline then defined by voxel analysis using Imaris software. (B) The center of distinct ATG8 puncta (bright green dots) are labeled using the option ‘Add new measurement points’ in the Imaris “spots” subroutine. (C) Distances from the hemozoin center to the ATG8 puncta centers are then computed (white lines) and data collated in excel.

**Figure 6:**
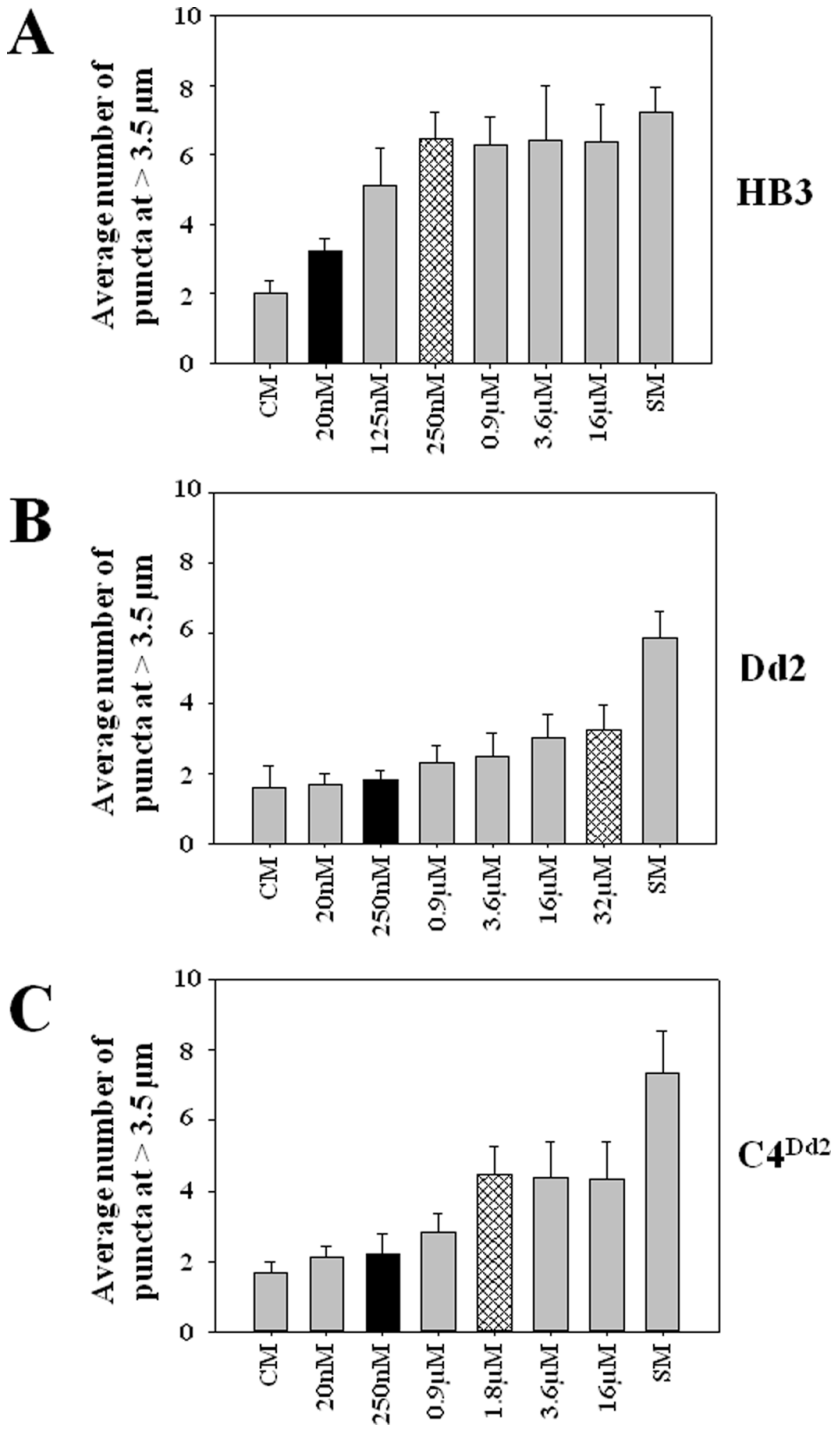
Quantified PfATG8 puncta distribution for synchronized trophozoite parasites. Shown are CQS HB3 (top) CQR Dd2 (middle) and transfectant C4^Dd2^ (bottom) under different conditions. Far left, “CM” = control culture conditions, far right, “SM” = iRBC in starvation media for 6 hr. In between are puncta quantified for iRBC treated for 6 hrs with the indicated [CQ]. Black bars in each panel denote 2×IC_50_ [CQ] for the strain, hashed bars denote 2×LD_50_ [CQ] for the strain. Data are the average of at least 20 iRBC, +/− s.d., and puncta that are ≥3.5 µm from DV hemozoin optical density are plotted. The three phenotypes are distinct, as evidenced by statistical comparison of results upon 250 nM and 3.6 µM treatments common to all three strains; for 3.6 µM data, HB3 vs Dd2, HB3 vs C4Dd2, and Dd2 vs C4Dd2, P value <0.05 in each case. For 250 nM data, HB3 vs Dd2 or HB3 vs C4Dd2, P value <0.05, but Dd2 vs C4Dd2>0.05. That is, as proposed in the text, C4Dd2 is intermediate relative to HB3 and Dd2: both Dd2 and C4Dd2 differ from HB3, but C4Dd2 shows behavior similar to Dd2 at lower dose CQ, but different behavior vs Dd2 at higher dose.

To quantify this behavior, we devised a method based on spinning disk confocal microscopy and 3D Imaris rendering of z stacks to plot radial distributions of PfATG8 puncta relative to hemozoin optical density (Fig. 5). Essentially, very optically dense hemozoin within the DV is used to define a “center” point of reference for the iRBC parasite (Fig. 5 left) and distinct, clearly defined spots of ATG8 fluorescence (Fig. 5 middle, see also Scheme S1) are then measured for their relative distance (x,y,z) from the center of hemozoin optical density (white lines, Fig. 5 right). Using these methods, we quantified PfATG8 puncta abundance at >3.5 µm for CQS HB3, CQR Dd2 and C4^Dd2^ parasites +/− starvation and across a range of bolus CQ dosages (Fig. 6). These puncta distributions show that starvation produces similar number and similar radially distributed patterns of peripheral PfATG8 puncta for CQS and CQR parasites (compare far left and far right bars in each panel Fig. 6), but that 2×CQS LD_50_ dose of CQ (250 nM) only produces high numbers of distal radially - distributed puncta for CQS strain HB3 (Fig. 6A top), not for CQR strain Dd2 (Fig. 6B middle). C4^Dd2^ transfectants, wherein CQR is mediated solely by allelic exchange with CQR associated mutant *pfcrt*, show PfATG8 behavior that is intermediate relative to CQS HB3 and CQR Dd2 (Fig. 6C bottom), and again, Dd2 shows a muted ATG8 response (hashed bars indicate 2×LD_50_ dose for each strain).

**Figure 7:**
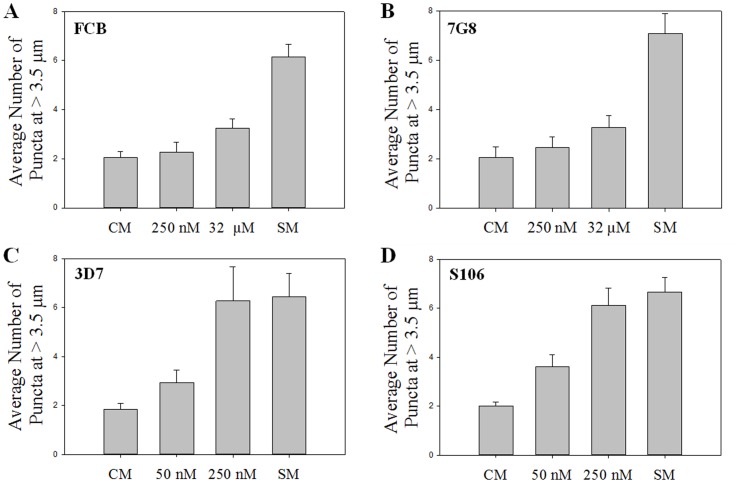
Quantified PfATG8 puncta at ≥3.5 µm from hemozoin for synchronized trophozoites. Two additional CQR and two additional CQS strains are analyzed. Shown are puncta counts for at least 20+/− s.d. grown under control conditions (“CM”, left side each panel), upon starvation (SM; far right, each panel) and upon dosing for 6 hrs with either 2×IC_50_ or 2×LD_50_ concentrations of CQ (50 nM and 250 nM for CQS strains, and 250 nM and 32 µM for CQR strains, respectively).

We further tested the association between peripherally distributed PfATG8– positive puncta and CQR status by examining 4 other well-characterized strains, two CQS (strains 3D7 and Sudan 106) and two CQR (strains FCB and 7G8). Upon starvation for 6 hr, all 4 strains distribute PfATG8– positive puncta in a peripheral fashion similar to starved HB3, Dd2, and C4Dd2 (Fig. 7). However, as shown in Fig. 7, only the CQR strains show no increased peripheral puncta at 250 nM CQ (2×CQS LD_50_) whereas both CQS strains do. Also, even though 2×CQR LD_50_ (32 µM) induces formation of peripheral puncta for the CQR strains, similar to CQR strain Dd2 (Fig. 6), the numbers are reduced relative to 2×CQS LD_50_ treated CQS. To summarize, 3 CQR laboratory strains behave similarly, and are distinct from all 3 CQS strains examined. In terms of statistical significance of these data, P<0.01 when puncta produced upon 250 nM CQ exposure for any CQS strain (HB3, 3D7, S106) is compared vs 250 nM CQ puncta data for any CQR strain (Dd2, FCB, 7G8). However, P values are >0.4 when any two CQR or any two CQS strains treated with the same dose are compared. That is, no CQR strains are significantly different from each other, no CQS strains are significantly different from each other, but all CQR strains show statistically significant differences in PfATG8 redistribution vs all CQS strains. This suggests that redistribution of PfATG can serve as a marker to quantify sensitivity to cytocidal CQ.

We next inventoried sequences of all *P. falciparum* orthologues of autophagy pathway proteins that we can identify at this time (see also [50]) using available genomes, and note that a number of putative orthologues of key autophagy proteins are mutated in Dd2 (CQR) vs HB3 and 3D7 (CQS) parasites (see Discussion). For example, a recently identified *P. falciparum* PI3’ kinase (PF3D7_0515300; [51]) appears to us to be a Vps34 orthologue, based on its very high identity to ScVps34 (E-value = 1×10^−73^; homology index score = 567). A similar conclusion was also reached by Kitamura *et al.* [47]. During autophagy in other eukaryotic cells, Vps34 produces copious PI3P at the developing autophagosome, where ATG8 protein also localizes. PfVps34 from CQR strain Dd2 shows point mutations and deletions relative to CQS strains HB3 and 3D7 that could conceivably affect function or regulation, as described in Discussion. Quite interestingly then, this enzyme has recently been localized to near Maurer’s cleft organelles [51], very similar to the PfATG8 relocalization upon starvation that is reported here. It remains to be determined if these additional autophagy gene mutations uniformly segregate with CQR^CC^ phenomena for geographically distinct isolates of CQR *P. falciparum*. Importantly, LD_50_ quantification for these isolates will need to be performed, which will require the ability to culture these isolates over long time periods.

## Discussion

Data in this paper reveal three important concepts:

1. Mutant PfCRT protein, while responsible for the majority of the shift in CQ IC_50_ that has traditionally characterized CQR *P. falciparum* strains Dd2 and 7G8 ([30], and present data), is only responsible for 10–20% of the shift in CQ LD_50_ for the same strains of CQR parasites. PfCRT is thought to confer CQR^CS^ by reducing access of CQ to DV – localized heme target and thereby reduce the ability of the drug to inhibit heme – hemozoin biomineralization [11], [29]. However, recent data [17] shows that DV levels of CQ are not necessarily correlated with CQ LD_50_. In addition, other recent work [18], [19] shows that the ability of CQ or QN analogues to inhibit hemozoin formation correlates with their IC_50_ activity, but not with their LD_50_ activity. Thus our observations are not as surprising as they might initially appear, and one important implication is that targets in addition to inhibition of hemozoin crystallization are likely relevant for the cytocidal activity of quinoline antimalarial drugs.
2. LD_50_– directed Quantitative trait loci (QTL) analysis in the well-characterized HB3 × Dd2 genetic cross shows that the chr5 segment previously identified as modulating PfCRT mediated CQR^CS^ does not contribute to CQR^CC^. Other distinct differences in genetic architecture include two novel loci, on chr6 and chr8, associated with elevated LD_50_. These loci are enriched for genes that encode proteins linked to proteosome and autophagy pathways, but no genes encoding metacaspases or other proteins that regulate apoptosis are found in the loci (Tables S2– S7). A number of recent observations suggest interesting cross talk between proteasome and autophagy pathways (e.g. [52], [53]), thus enrichment for both processes in the LD_50_ loci is intriguing.
3. Staining for a definitive marker of autophagy (ATG8 protein puncta) shows cell death from LD_50_ CQ treatment is linked to an autophagy – like process and that this process is altered in CQR parasites. Interestingly, we find that starvation and CQ – induced death lead to accumulation of PfATG8– positive puncta to near Maurer’s clefts (MC). These poorly understood organelles are known to be involved in export of proteins from the parasite to the red cell plasma membrane but they are also believed to have several other functions, including vesicle fusion [54].

To our knowledge, only three studies have attempted to distinguish CQ cytostatic and cytocidal activities *in vitro* [5], [6], [55] but only recently have LD_50_ been quantified [6], allowing QTL analyses as presented here. All previous cellular quantification of antimalarial drug resistance phenomena of which we are aware has been solely via IC_50_ ratios. IC_50_ does not *a priori* define cytocidal (parasite kill) potency; formally it defines growth inhibition potency vs mass populations of parasites. It is certainly true that killing some parasites over time with a drug prevents growth seen in control cultures, but it can also be true that a drug in continuous culture does not kill parasites, but merely slows the cell cycle, changes multiplicity of schizogony, or has other effects that yield IC_50_ of a given value for mass populations of parasites [7]. CQ appears to exhibit both dose dependent (“C_max_”) ([7] and this work) and time dependent (“T_MIC_”) [56] parasite killing. In studies that report IC_90_ data there may be greater “mixing” between CQ cytostatic and cytocidal effects relative to studies that report IC_50_ data. In this paper, we have made the first attempt to our knowledge to more fully separate CQ IC_50_ and LD_50_ phenomena for progeny of the HB3×Dd2 cross.

We previously found that some strains are much more CQR^CC^ than CQR^CS^ [6], and that the rank order of LD_50_ and IC_50_ for different drugs varies (meaning the drugs to which a parasite shows the highest IC_50_ are not necessarily the drugs to which it shows the highest LD_50_ [6]). Plotting CQ IC_50_ [4] vs CQ LD_50_ [Table S1] for HB3×Dd2 progeny yields poor correlation (r^2^<0.4; not shown). Also, a recent study shows that CQ transport ability for 13 mutant PfCRT isoforms found in 13 different CQR strains does not correlate with CQ IC_50_ for those strains [57]. Considering these observations along with the current data suggests that parasite resistance to cytocidal effects of CQ is influenced by additional genetic or physiological events, along with PfCRT mutations. Our initial analysis suggests these events include alterations in a novel pathway showing some similarities to autophagy.

In all eukaryotic cell types examined, the re distribution of ATG8 protein to more widely disbursed puncta marks the induction of autophagy by starvation [46] **.** In all other examples, the membranes to which ATG8 is routed are synthesizing double membraned autophagosomes and copius phosphatidyl inositol 3′ phosphate (PI3P) via Vps34. It is striking then that previous work completely unrelated to the present study places PfVps34 near MC, similar to our localization for some re – routed PfATG8 [51]. In all eukaryotic cell types examined, autophagosomes containing high levels of ATG8 and PI3P lipid then engulf cytosolic or organellar targets, fuse with lysosomes, and the contents are then degraded, serving as nutrient pools that temporarily keep the starving cell alive. In the case of *P. falciparum*, the parasite trophozoite undergoes heightened accumulation of PfATG8– associated vesicles at or near the MC upon starvation and cytocidal CQ treatment. The parasite trophozoite does not appear to engulf and degrade its sole mitochondrion to provide additional food upon starvation. Indeed, starvation induced mitochondrial fragmentation by autophagy in *T. gondii* causes cell death [31]. Instead, the unique properties of the RBC, which is devoid of de novo biosynthetic activity as a host cell, necessitates enhanced endocytosis to acquire extracellular food. We suggest that under starvation pressure the parasite up regulates additional endocytosis from the red cell cytosol using (at least in part) the vesicle formation and fusion functions of encoded autophagy machinery. This might be consistent with PfATG8 vesicles eventually interacting with the parasite DV, analogous to ATG8 positive vesicle fusion to lysosomes in other eukaryotes after they recruit nutritional “cargo”.

In yeast and higher eukaryotes, membrane association of ATG8 is mediated by lipidation. The terminal G residue of ATG8 that becomes lipidylated is blocked, necessitating proteolytic cleavage by ATG4 as a prelude to membrane association [46]. Notably however, *P. falciparum* PfATG8 terminates with G, thus ATG4 proteolysis to reveal G as in other species is absent, suggestive of constitutive membrane association [49]. In reality however, PfATG8 exists in both the unlipidated and lipidated forms (Fig. 2D) suggesting mechanisms other than ATG4 regulate PfATG8 dynamics. We propose that a low level of constitutively activated autophagy is present in iRBC parasites, and that CQR parasites have developed resistance to CQ induced perturbations in autophagy. Accumulation of PfATG8 puncta upon toxic CQ treatment is consistent with either upregulation of puncta formation or inhibition of autophagosome fusion, so CQR parasites could in theory have perturbations in either (or both) steps of the autophagy pathway.

Interestingly then, CQ is a known potent inhibitor of autophagy in other cell types. Its diprotic weak base character promotes profound accumulation in acidic compartments such as lysosomes, autophagosomes, and vacuoles. At doses that correspond to these LD_50_ CQ is known to block the fusion of autophagosomes with lysosomes/vacuoles and also raises the pH of these compartments, thereby inhibiting processes that require acidic pH (e.g. intra lysosomal proteolysis ([42], [44] and references within)). A few molecular possibilities specific to *P. falciparum* are that LD_50_ dosages of CQ (i) block the fusion of endocytic vesicles carrying hemoglobin to the DV (in fact, a somewhat overlooked paper [58] shows that CQ LD_50_ doses do indeed lead to a buildup of undigested Hb trapped within arrested parasite vesicles), (ii) inhibit falcipain and plasmepsin activity by raising the pH in endocytic vesicles and/or the DV, (iii) inhibit fusion of autophagosomes and/or other vesicles with their target organelles. Other lysosomotropic agents would be expected to mimic this CQ pharmacology. Interestingly then, certain alkaloids that inhibit autophagy also show antimalarial activity [59], [60]. One example is voacamine, a tertiary alkaloid isolated from *Peschiera fuchsiaefolia* stem bark that shows good antimalarial activity (238 ng/mL vs strain D6 and 290 ng/mL vs strain W2), and which has also been reported to chemosensitize MDR cancer cells in an autophagy-dependent manner [61]. Overall, since *P. falciparum* has been subjected to decades of cytocidal CQ selective pressure, it is logical that the parasites would evolve resistance to CQ autophagy inhibition.

With regard to the starvation effects that we observe, work in the Goldberg laboratory has shown that *P. falciparum* meets its amino acid requirements by a combination of hemoglobin degradation and uptake of free amino acids from the medium [62]–[64]. When some extracellular amino acids are removed, the parasite responds by up – regulating additional hemoglobin transport and degradation; hemoglobin, however, lacks the essential amino acid Ile, so parasite survival is conditional under these circumstances [63]. Conversely, if the hemoglobin pathway is inhibited, the parasite survives by acquiring additional amino acids from the extracellular medium. If Ile is withdrawn the parasite can enter a hibernatory state [64]. These observations suggest that (i) malaria parasites are able to sense amino acid levels in the medium and (ii) they possess a system that can respond to the lack of some extracellular amino acids by regulating intracellular transport to the vacuole. During starvation induced autophagy, other eukaryotic cells respond to low amino acid levels in the medium by trapping cytosolic material in transport vesicles, which will eventually fuse and release cargo into a lysosome or vacuole to then be digested to amino acids. Although for intraerythrocytic *P. falciparum* the “cargo” is presumably within the host cell cytosol, starvation induced autophagy reported here could be somewhat reminiscent of elevated hemoglobin endocytosis in *P. falciparum*.

We wondered if autophagy genes in the identified chr6/chr8 loci might be hinting at mutations in other Pf autophagy gene (PfATG gene) orthologues for CQR parasites. A partial inventory of PfATG gene orthologues has been published [50] but does not include important co factors such as Vps34, Pex14, Vps15, etc. We re – queried the Pf genome with a more complete set of 42 autophagy related genes. Remarkably, a number of candidate orthologues (16 of 42) lie within QTL loci previously associated with drug resistance phenomena, or within “eQTL” gene sets that are up/down regulated in trans by resistance associated eQTL [35]. Also, after alignment of Dd2 (CQR) vs HB3 and 3D7 (CQS) alleles via Broad Institute data (http://www.broadinstitute.org/), we find that many candidate PfATG gene orthologues are mutated in the Dd2 CQR genome relative to 3D7 or HB3 CQS. One in particular is candidate PfVps34 (PF3D7_0515300; E-value 10^−73^ and homology index score 567 relative to ScVps34) which encodes K at codon 423 for Dd2 in place of Q found for HB3 and 3D7, deletion of a N residue found at position 578 for HB3 and 3D7, and a deletion of 24 codons relative to HB3 and 3D7 (from codon 931 to 955). Interestingly, this deletion is within what we predict is a C2 regulatory domain that presumably binds Ca^2+^; a similar C2 domain prediction for this protein has also been made by two other groups [51], [65]. Since a clear TOR orthologue is missing within the *P. falciparum* genome [50] altered regulation via mutant PfVps34 is one possibility for the altered cascade seen in CQR strain Dd2.

Regardless, we predict that multiple routes to dysregulated autophagy will be found for various strains and isolates of CQR^CC^ *P. falciparum*, analogous to how multiple routes to dysregulated programmed cell death are found in various multidrug resistant tumor cell lines [16]. At LD_50_ doses, via its well known lysosomotropic behavior [58], [66] CQ will inhibit vesicle formation and vesicle fusion, two processes that are essential to autophagy. Since these processes are controlled by a number of proteins, many mutations are possible for altering CQ response.

In sum, we find that mutant PfCRT protein confers the majority of CQR^CS^ as defined by CQ IC_50_ shift, but only partial CQR^CC^ as defined by LD_50_ shift. Not surprisingly then, our data also show that distinct genetic architecture is associated with CQR^CS^ vs CQR^CC^, that a specialized *P. falciparum* autophagy cascade is induced by LD_50_ doses of CQ but not IC_50_ doses of the drug, and that altered regulation of this unique autophagy – like process is present in CQR^CC^ parasites. Consistent with a clear role for autophagy in cell death for other apicomplexa [31], [45], altered PfATG8 redistribution indicative of a novel autophagy – like cascade appears to be associated with CQR^CC^ for intraerythrocytic *P. falciparum* trophozoites. Elucidating additional molecular events controlling perturbation of autophagy signaling for CQR *P. falciparum*, potentially involving at least two dozen autophagy gene orthologues [50], should prove to be a fertile area of future research. Among additional data needed to elucidate the pathway further are precise measurements of ATG8 puncta flux, meaning the rate of ATG8 vesicle production vs presumed fusion with either MC and perhaps the DV.

Finally, regardless the exact role of the novel autophagy – like process for iRBC trophozoites that we describe here, we propose that CQR^CC^ requires prior acquisition of CQR^CS^, meaning that parasites must first be able to survive lower IC_50_ dose via PfCRT mutations, before they can survive higher LD_50_ dose via acquiring additional autophagy mutations. Precise measurements of IC_50_ vs LD_50_ phenomena among geographically diverse isolates of CQR *P. falciparum* will further test this hypothesis. Also, since PvCRT mutations have not been found for CQR *P. vivax*, it should prove interesting to investigate the status of genes in the chr6 and chr8 loci for CQR *P. vivax* isolates, as well as the formation of PvATG8 positive structures in response to CQ treatment.

## Supporting Information

Figure S1 **Late trophozoite/early schizont stained with anti-ATG8 (green) and anti-apicoplast (red) antibodies.** Some overlap between ATG8 and apicoplast localized ACP (D) suggests partial (but not complete, ∼25%) co – localization of PfATG8 and apicoplast as previously suggested [46]. Scale bar = 5 µm.
(DOC)

Figure S2 **CQS (HB3, A) and CQR (Dd2, B) parasites treated with IC_50_ (top row each panel) and 2× IC_50_ (bottom row) doses of CQ (10, 20 nM and 125, 250 nM, respectively) for 48 hr.** Parasites were then stained for ATG8 (green) and with DAPI (blue), as described in Methods. Very few peripherally disposed PfATG8 puncta are observed, supporting the conclusion that puncta formation is associated with LD_50_, but not IC_50_ doses of CQ. Scale bar = 5 µm.
(DOC)

Table S1 **LD_50_ values for the HB3×Dd2 cross progeny.**
(DOC)

Table S2 **All genes within the chr6 LD_50_ locus.**
(DOC)

Table S3 **All genes within the LD_50_ chr6×chr8 interaction.**
(DOC)

Table S4 **GO enriched molecular functions for the chr6 LD_50_ locus.**
(DOC)

Table S5 **GO enriched biological processes for the LD_50_ chr6 locus.**
(DOC)

Table S6 **GO enriched molecular functions for the LD_50_ chr6×chr8 interaction.**
(DOC)

Table S7 **GO enriched biological processes for the LD_50_ chr6×chr8 interaction.**
(DOC)

Scheme S1 **Cartoon representation of 3D puncta quantification method used in the present work.** Hemozoin density (red “x”) is used as the origin, and distances toPfATG8 positive puncta are defined for the reconstructed confocal z stack of images, relative to hemozoin, using 3D Cartesian (x,y,z) coordinates. The cartoon shows an abbreviated depiction of 3 SDCM “slices”, but as described in methods the z stack data set for each cell is a fully assembled, iteratively deconvolved 3D image constructed from approximately 15–20 z slices (see [35] for additional detail).
(DOC)

## References

[1] Cheeseman IH, Miller BA, Nair S, Nkhoma S, Tan A, et al (2012) A major genome region underlying artemisinin resistance in malaria. Science 336: 79–82. 22491853 10.1126/science.1215966 PMC3355473

[2] Plowe CV, Cortese JF, Djimde A, Nwanyanwu OC, Watkins WM, et al (1997) Mutations in Plasmodium falciparum dihydrofolate reductase and dihydropteroate synthase and epidemiologic patterns of pyrimethamine-sulfadoxine use and resistance. J Infect Dis. 176: 1590–1596. 10.1086/514159 9395372

[3] Wang P, Read M, Sims PF, Hyde JE (1997) Sulfadoxine resistance in the human malaria parasite Plasmodium falciparum is determined by mutations in dihydropteroate synthetase and an additional factor associated with folate utilization. Mol Microbiol. 23: 979–86. 10.1046/j.1365-2958.1997.2821646.x 9076734

[4] Fidock DA, Nomura T, Talley AK, Cooper RA, Dzekunov SM, et al (2000) Mutations in the P. falciparum digestive vacuole transmembrane protein PfCRT and evidence for their role in chloroquine resistance. Mol Cell. 4: 861–871. 10.1016/s1097-2765(05)00077-8 PMC2944663 11090624

[5] Young RD, Rathod PK (1993) Clonal viability measurements on Plasmodium falciparum to assess in vitro schizonticidal activity of leupeptin, chloroquine, and 5-fluoroorotate. Antimicrob Agents Chemother. 37: 1102–1107. 10.1128/aac.37.5.1102 PMC187909 8517698

[6] Paguio MF, Bogle KL, Roepe PD (2011) Plasmodium falciparum resistance to cytocidal versus cytostatic effects of chloroquine. Mol Biochem Parasitol. 178: 1–6. 10.1016/j.molbiopara.2011.03.003 PMC3101316 21470564

[7] Gligorijevic B, Purdy K, Elliott DA, Cooper RA, Roepe PD (2008) Stage independent chloroquine resistance and chloroquine toxicity revealed via spinning disk confocal microscopy. Mol Biochem Parasitol. 159: 7–23. 10.1016/j.molbiopara.2007.12.014 PMC2440633 18281110

[8] Ferdig MT, Cooper RA, Mu J, Deng B, Joy DA, et al (2004) Dissecting the loci of low-level quinine resistance in malaria parasites. Mol Microbiol. 52: 985–997. 10.1111/j.1365-2958.2004.04035.x 15130119

[9] Sá JM, Twu O, Hayton K, Reyes S, Fay MP, et al (2009) Geographic patterns of Plasmodium falciparum drug resistance distinguished by differential responses to amodiaquine and chloroquine. Proc Natl Acad Sci U S A. 106: 18883–18889. 10.1073/pnas.0911317106 PMC2771746 19884511

[10] Patel JJ, Thacker D, Tan JC, Pleeter P, Checkley L, et al (2010) Chloroquine susceptibility and reversibility in a Plasmodium falciparum genetic cross Mol Microbiol. 78: 770–787. 10.1111/j.1365-2958.2010.07366.x PMC3091165 20807203

[11] Roepe PD (2011) PfCRT-Mediated Drug Transport in Malarial Parasites. Biochemistry. 50: 163–171. 10.1021/bi101638n PMC3123679 21142008

[12] Mäser P, Wittlin S, Rottmann M, Wenzler T, Kaiser M, et al (2012) Antiparasitic agents: new drugs on the horizon. Curr Opin Pharmacol. 12: 562–566. 10.1016/j.coph.2012.05.001 22652215

[13] Salako LA, Aderounmu AF, Walker O (1987) Influence of route of administration on the pharmaco-kinetics ofchloroquine and desethylchloroquine. Bull World Health Organ. 65: 47–50. PMC2490847 3495365

[14] Hodel EM, Zanolari B, Mercier T, Biollaz J, Keiser J, et al (2009) A single LC-tandem mass spectrometry method for the simultaneous determination of 14 antimalarial drugs and their metabolites in human plasma. J Chromatogr B Analyt Technol Biomed Life Sci. 877: 867–886. 10.1016/j.jchromb.2009.02.006 19249251

[15] Khalil IF, Alifrangis M, Recke C, Hoegberg LC, Ronn A, et al (2011) Development of ELISA-based methods to measure the anti-malarial drug chloroquine in plasma and in pharmaceutical formulations. Malar J. 10: 249–253. 10.1186/1475-2875-10-249 PMC3170649 21864375

[16] Baguley BC (2010) Multiple drug resistance mechanisms in cancer. Mol Biotechnol. 46: 308–316. 10.1007/s12033-010-9321-2 20717753

[17] Cabrera M, Paguio MF, Xie C, Roepe PD (2009) Reduced digestive vacuolar accumulation of chloroquine is not linked to resistance to chloroquine toxicity. Biochemistry 48: 11152–11164. 19883122 10.1021/bi901765v PMC2788207

[18] Gorka AP, Alumasa JN, Sherlach KS, Jacobs LM, Nickley KB, et al (2013a) Cytostatic vs Cytocidal Activities of Chloroquine Analogues and Inhibition of Hemozoin Crystal Growth. Antimicro. Agents and Chemotherapy. 57: 356–364. 10.1128/AAC.01709-12 PMC3535939 23114783

[19] Gorka AP, Sherlach KS, de Dios AC, Roepe PD (2013b) Relative to Quinine and Quinidine, their 9-epimers exhibit decreased cytostatic activity and altered heme binding, but similar cytocidal activity vs P. falciparum. Antimicro. Agents and Chemotherapy. 57: 365–374. 10.1128/AAC.01234-12 PMC3535971 23114754

[20] Gorka AP, de Dios A, Roepe PD (2013c) Quinoline Drug-Heme Interactions and Implications for Antimalarial Cytostatic versus Cytocidal Activities. J Med Chem. 2013 Apr 29. [Epub ahead of print, PMID: 23586757] 10.1021/jm400282d 23586757

[21] Sinai AP, Roepe PD (2012) Autophagy in Apicomplexa: a life sustaining death mechanism? Trends Parasitol. 28: 358–364. 10.1016/j.pt.2012.06.006 PMC4354876 22819059

[22] Totino PR, Daniel-Ribeiro CT, Corte-Real S, de Fátima Ferreira-da-Cruz M (2008) Plasmodium falciparum: erythrocytic stages die by autophagic-like cell death under drug pressure. Exp Parasitol. 118: 478–86. 10.1016/j.exppara.2007.10.017 18226811

[23] Meslin B, Beavogui AH, Fasel N, Picot S (2011) Plasmodium falciparum metacaspase PfMCA-1 triggers a z-VAD-fmk inhibitable protease to promote cell death. PLoS One. 6: e23867. 10.1371/journal.pone.0023867 PMC3157471 21858231

[24] Suradji EW, Hatabu T, Kobayashi K, Yamazaki C, Abdulah R, et al (2011) Selenium-induced apoptosis-like cell death in Plasmodium falciparum. Parasitology. 19: 1–11. 10.1017/S0031182011001399 21854677

[25] Ch’ng JH, Kotturi SR, Chong AG, Lear MJ, Tan KS (2010) A programmed cell death pathway in the malaria parasite Plasmodium falciparum has general features of mammalian apoptosis but is mediated by clan CA cysteine proteases. Cell Death Dis. 1: e26. 10.1038/cddis.2010.2 PMC3032337 21364634

[26] Nyakeriga AM, Perlmann H, Hagstedt M, Berzins K, Troye-Blomberg M, et al (2006) Drug-induced death of the asexual blood stages of Plasmodium falciparum occurs without typical signs of apoptosis. Microbes Infect. 8: 1560–1568. 10.1016/j.micinf.2006.01.016 16702009

[27] Sanchez CP, Mayer S, Nurhasanah A, Stein WD, Lanzer M (2011) Genetic linkage analyses redefine the roles of PfCRT and PfMDR1 in drug accumulation and susceptibility in Plasmodium falciparum. Mol Microbiol. 82: 865–878. 10.1111/j.1365-2958.2011.07855.x 21999470

[28] Cooper RA, Ferdig MT, Su XZ, Ursos LM, Mu J, et al (2002) Alternative mutations at position 76 of the vacuolar transmembrane protein PfCRT are associated with chloroquine resistance and unique stereospecific quinine and quinidine responses in Plasmodium falciparum. Mol Pharmacol. 61: 35–42. 10.1124/mol.61.1.35 11752204

[29] Ecker A, Lehane AM, Clain J, Fidock DA (2012) PfCRT and its role in antimalarial drug resistance. Trends Parasitol. pii: S1471–4922. 10.1016/j.pt.2012.08.002 PMC3478492 23020971

[30] Sidhu AB, Verdier-Pinard D, Fidock DA (2002) Chloroquine resistance in Plasmodium falciparum malaria parasites conferred by pfcrt mutations. Science. 298: 210–213. 10.1126/science.1074045 PMC2954758 12364805

[31] Ghosh D, Walton JL, Roepe PD, Sinai AP (2012) Establishment of autophagy as a cell death mechanism in Toxoplasma gondii Cell Microbiol. 4: 589–607. 10.1111/j.1462-5822.2011.01745.x PMC3302960 22212386

[32] Bennett TN, Paguio M, Gligorijevic B, Seudieu C, Kosar AD, et al (2004) Novel, rapid, and inexpensive cell-based quantification of antimalarial drug efficacy. Antimicrob Agents Chemother. 48: 1807–1810. 10.1128/AAC.48.5.1807-1810.2004 PMC400551 15105139

[33] Reilly Ayala H, Wacker MA, Siwo G, Ferdig MT (2011) Quantitative trait loci mapping reveals candidate pathways regulating cell cycle duration in Plasmodium falciparum. BMC Genomics. 11: 577–587. 10.1186/1471-2164-11-577 PMC3091725 20955606

[34] Huang DW, Sherman BT, Lempicki RA (2009) Systematic and integrative analysis of large gene lists using DAVID Bioinformatics Resources. Nature Protoc. 4(1): 44–57. 10.1038/nprot.2008.211 19131956

[35] Gonzales JM, Patel JJ, Ponmee N, Jiang L, Tan A, et al (2008) Regulatory hotspots in the malaria parasite genome dictate transcriptional variation. PLoS Biol. 30 6(9): e238. 10.1371/journal.pbio.0060238 PMC2553844 18828674

[36] Gligorijevic B, McAllister R, Urbach JS, Roepe PD (2006) Spinning disk confocal microscopy of live, intraerythrocytic malarial parasites. 1. Quantification of hemozoin development for drug sensitive versus resistant malaria. Biochemistry. 45: 12400–12410. 10.1021/bi061033f 17029396

[37] Waller KL, Muhle RA, Ursos LM, Horrocks P, Verdier-Pinard D, et al (2003) Chloroquine resistance modulated in vitro by expression levels of the Plasmodium falciparum chloroquine resistance transporter. J. Biol. Chem. 278: 33593–33601. 10.1074/jbc.M302215200 12813054

[38] Wilson CM, Serrano AE, Wasley A, Bogenschutz MP, et al (1989) Amplification of a gene related to mammalian mdr genes in drug-resistant Plasmodium falciparum. Science. 244: 1184–1186. 10.1126/science.2658061 2658061

[39] Reed MB, Saliba KJ, Caruana SR, Kirk K, Cowman AF (2000) Pgh1 modulates sensitivity and resistance to multiple antimalarials in Plasmodium falciparum. Nature. 403: 906–9. 10.1038/35002615 10706290

[40] Amoah LE, Lekostaj JK, Roepe PD (2007) Heterologous expression and ATPase activity of mutant versus wild type PfMDR1 protein. Biochemistry 46: 6060–6073. 17469853 10.1021/bi7002026

[41] Pleeter P, Lekostaj JK, Roepe PD (2010) Purified Plasmodium falciparum multi-drug resistance protein (PfMDR 1) binds a high affinity chloroquine analogue. Mol Biochem Parasitol. 173: 158–161. 10.1016/j.molbiopara.2010.05.012 PMC2906614 20546803

[42] Notte A, Leclere L, Michiels C (2011) Autophagy as a mediator of chemotherapy-induced cell death in cancer. Biochem Pharmacol. 82: 427–434. 10.1016/j.bcp.2011.06.015 21704023

[43] Platini F, Pérez-Tomás R, Ambrosio S, Tessitore L (2010) Understanding autophagy in cell death control. Curr Pharm Des. 16: 101–13. 10.2174/138161210789941810 20214621

[44] Gozuacik G, Kimchi K (2007) Autophagy and Cell death Curr. Topics in Dev Biol 78: 217–245. 10.1016/S0070-2153(06)78006-1 17338918

[45] Lavine MD, Arrizabalaga G (2012) Analysis of monensin sensitivity in Toxoplasma gondii reveals autophagy as a mechanism for drug induced death. PLoS One. 7(7): e42107 10.1371/journal.pone.0042107 PMC3405052 22848721

[46] Shpilka T, Weidberg H, Pietrokovski S, Elazar Z (2011) ATG8: An Autophagy – Related Ubiquitin – Like Protein Family. Genome Biology 12: 226–238. 21867568 10.1186/gb-2011-12-7-226 PMC3218822

[47] Kitamura K, Kishi-Itakura C, Tsuboi T, Sato S, Kita K, et al (2012) Autophagy-related Atg8 localizes to the apicoplast of the human malaria parasite Plasmodium falciparum. PLoS One 7 8: e42977 doi:10.1371 22900071 10.1371/journal.pone.0042977 PMC3416769

[48] Matsui A, Kamada Y, Matsuura A (2013) The role of autophagy in genome stability through suppression of abnormal mitosis under starvation. PLoS Genet. 9(1): e1003245 10.1371/journal.pgen.1003245 PMC3561091 23382696

[49] Dotiwala F, Eapen VV, Harrison JC, Arbel-Eden A, Ranade V, et al (2013) DNA damage checkpoint triggers autophagy to regulate the initiation of anaphase. Proc Natl Acad Sci U S A. 110: E41–49. 10.1073/pnas.1218065109 PMC3538254 23169651

[50] Brennand A, Gualdrón-López M, Coppens I, Rigden DJ, Ginger ML, et al (2011) Autophagy in parasitic protists: unique features and drug targets. Mol Biochem Parasitol. 177: 83–99. 10.1016/j.molbiopara.2011.02.003 21315770

[51] Vaid A, Ranjan R, Smythe WA, Hoppe HC, Sharma P (2010) PfPI3K, a phosphatidylinositol-3 kinase from Plasmodium falciparum, is exported to the host erythrocyte and is involved in hemoglobin trafficking. Blood. 115: 2500–2507. 10.1182/blood-2009-08-238972 PMC2918364 20093402

[52] Taguchi K, Fujikawa N, Komatsu M, Ishii T, Unno M, et al (2012) Keap1 degradation by autophagy for the maintenance of redox homeostasis. Proc Natl Acad Sci U S A. 109(34): 13561–6. 10.1073/pnas.1121572109 PMC3427110 22872865

[53] Lin M, Chandramani-Shivalingappa P, Jin H, Ghosh A, Anantharam V, et al (2012) Methamphetamine-induced neurotoxicity linked to ubiquitin-proteasome system dysfunction andautophagy-related changes that can be modulated by protein kinase C delta in dopaminergic neuronal cells. Neuroscience. 17 210: 308–32. 10.1016/j.neuroscience.2012.03.004 PMC3358550 22445524

[54] Lanzer M, Wickert H, Krohne G, Vincensini L, Braun Breton C (2006) Maurer’s clefts: A novel multi-functional organelle in the cytoplasm of Plasmodium falciparum-infected erythrocytes. Intl. J. Parasitol. 36: 23–36. 10.1016/j.ijpara.2005.10.001 16337634

[55] Richards WH, Maples BK (1979) Studies on Plasmodium falciparum in continuous cultivation. I. The effect of chloroquine and pyrimethamine on parasite growth and viability. Ann Trop Med Parasitol. 73: 99–108. 386970

[56] Bakshi RP, Nenortas E, Tripathi AK, Sullivan DJ, Shapiro TA (2013) Model System to Define Pharmacokinetic Requirements for Antimalarial Drug Efficacy. Science (Translational Medicine). In the press. 10.1126/scitranslmed.3006684 PMC3845824 24089407

[57] Baro NK, Callaghan PS, Roepe PD (2013) Function of resistance conferring Plasmodium falciparum chloroquine resistance transporter isoforms. Biochemistry. 52: 4242–4249. 10.1021/bi400557x PMC3703759 23688277

[58] Roberts L, Egan TJ, Joiner KA, Hoppe HC (2008) Differential effects of quinoline antimalarials on endocytosis in Plasmodium falciparum. Antimicrob Agents Chemother. 52: 1840–1842. 10.1128/AAC.01478-07 PMC2346632 18316523

[59] Federici E, Palazzino G, Nicoletti M, Galeffi C (1999) Antiplasmodial activity of the alkaloids of Peschiera fuchsiaefolia. 66: 93–95. 10.1055/s-0029-1243122 10705750

[60] Ramanitrahasimbola D, Rasoanaivo P, Ratsimamanga-Urverg S, Federici E, Palazzino G, et al (2001) Biological activities of the plant-derived bisindole voacamine with reference to malaria. Phythother. Res. 15: 30–33. 10.1002/1099-1573(200102)15:1<30::aid-ptr680>3.0.co;2-t 11180519

[61] Meschini S, Condello M, Marra M, Formisano G, Federici E, et al (2007) Autophagy-mediated chemosensitizing effect of the plant alkaloid voacamine on multidrug resistant cells. Toxicol. 21: 197–203. 10.1016/j.tiv.2006.09.007 17070665

[62] Liu J, Istvan ES, Gluzman IY, Gross J, Goldberg DE (2006) Plasmodium falciparum ensures its amino acid supply with multiple acquisition pathways and redundant proteolytic enzyme systems. Proc. Natl. Acad. Sci. 103: 8840–8845. 10.1073/pnas.0601876103 PMC1470969 16731623

[63] Istvan ES, Dharia NV, Bop SE, Gluzman I, Winzeler EA, et al (2011) Validation of isoleucine utilization targets in Plasmodium falciparum. Proc. Natl. Acad. Sci. 108: 1627–1632. 10.1073/pnas.1011560108 PMC3029723 21205898

[64] Babbitt SE, Altenhofen L, Cobbold SA, Istvan ES, Fennell C, et al.. (2012) Plasmodium falciparum responds to amino acid starvation by entering into a hibernatory state. Proc. Natl. Acad. Sci. USA in press. (early edition doi 10.1073). 10.1073/pnas.1209823109 PMC3511138 23112171

[65] Tawk L, Chicanne G, Dubremetz JF, Richard V, Payrastre B, et al (2010) Phosphatidylinositol 3-phosphate, an essential lipid in Plasmodium, localizes to the food vacuole membrane and the apicoplast. Eukaryotic Cell 9: 1519–1530. 20709789 10.1128/EC.00124-10 PMC2950420

[66] de Duve C, de Barsy T, Poole B, Trouet A, Tulkens P, et al (1974) Lysosomotropic agents. Biochem Pharmacol. 23: 2495–531. 10.1016/0006-2952(74)90174-9 4606365

