# Supplementary material for: A Process Similar to Autophagy Is Associated with Cytocidal Chloroquine Resistance in Plasmodium falciparum
Source: PLoS One. 2013 Nov 20;8(11):e79059. doi: 10.1371/journal.pone.0079059 (PMC3835802; doi:10.1371/journal.pone.0079059)
Supplement: Figure S1 — Late trophozoite/early schizont stained with anti-ATG8 (green) and anti-apicoplast (red) antibodies. Some overlap between ATG8 and apicoplast localized ACP (D) suggests partial (but not complete, ∼25%) co – localization of PfATG8 and apicoplast as previously suggested [46]. Scale bar = 5 µm. (DOC) [file pone.0079059.s001.doc]

**Figure S1. Trophozoite co-stained with anti-ATG8 (green) and anti-apicoplast (red) antibodies.**

Some overlap between ATG8 and apicoplast localized ACP (D) suggests partial (but not complete, ~ 25%) co – localization of PfATG8 and apicoplast as previously suggested [46]. Scale bar = 5µm. Spinning disk confocal images of trophozoite-infected erythrocyte fixed with 4% paraformaldehyde / 0.008% glutaraldehyde, treated with rat anti-ACP (apicoplast specific) and rabbit anti-TgATG8 primary antibodies (1:500), and stained with goat anti-rat AlexaFluor585 (B) and goat anti-rabbit DyLight488 (C) secondary antibodies (1:500). Imaging was carried out at 35% laser power and 200ms exposure time using available 491 and 561nm laser lines. Images were deconvolved using AutoQuantX2 and further processed with Imaris 7.5.2.

**
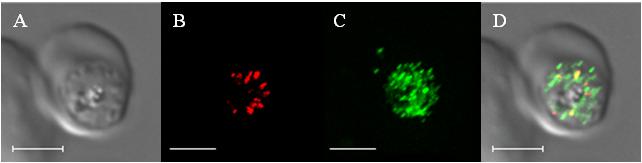
**
