# Supplementary material for: A Process Similar to Autophagy Is Associated with Cytocidal Chloroquine Resistance in Plasmodium falciparum
Source: PLoS One. 2013 Nov 20;8(11):e79059. doi: 10.1371/journal.pone.0079059 (PMC3835802; doi:10.1371/journal.pone.0079059)

**Figure S2. CQS (HB3, A) and CQR (Dd2, B) parasites treated with IC_50_ (top row each panel) and 2 x IC_50_ (bottom row) doses of CQ (10, 20 nM and 125, 250 nM, respectively) for 48 hr.** Parasites were then stained for ATG8 (green) and with DAPI (blue), as described in Methods. Very few peripherally disposed PfATG8 puncta are observed, supporting the conclusion that puncta formation is associated with LD_50_, but not IC_50_ doses of CQ. Scale bar = 5µm.


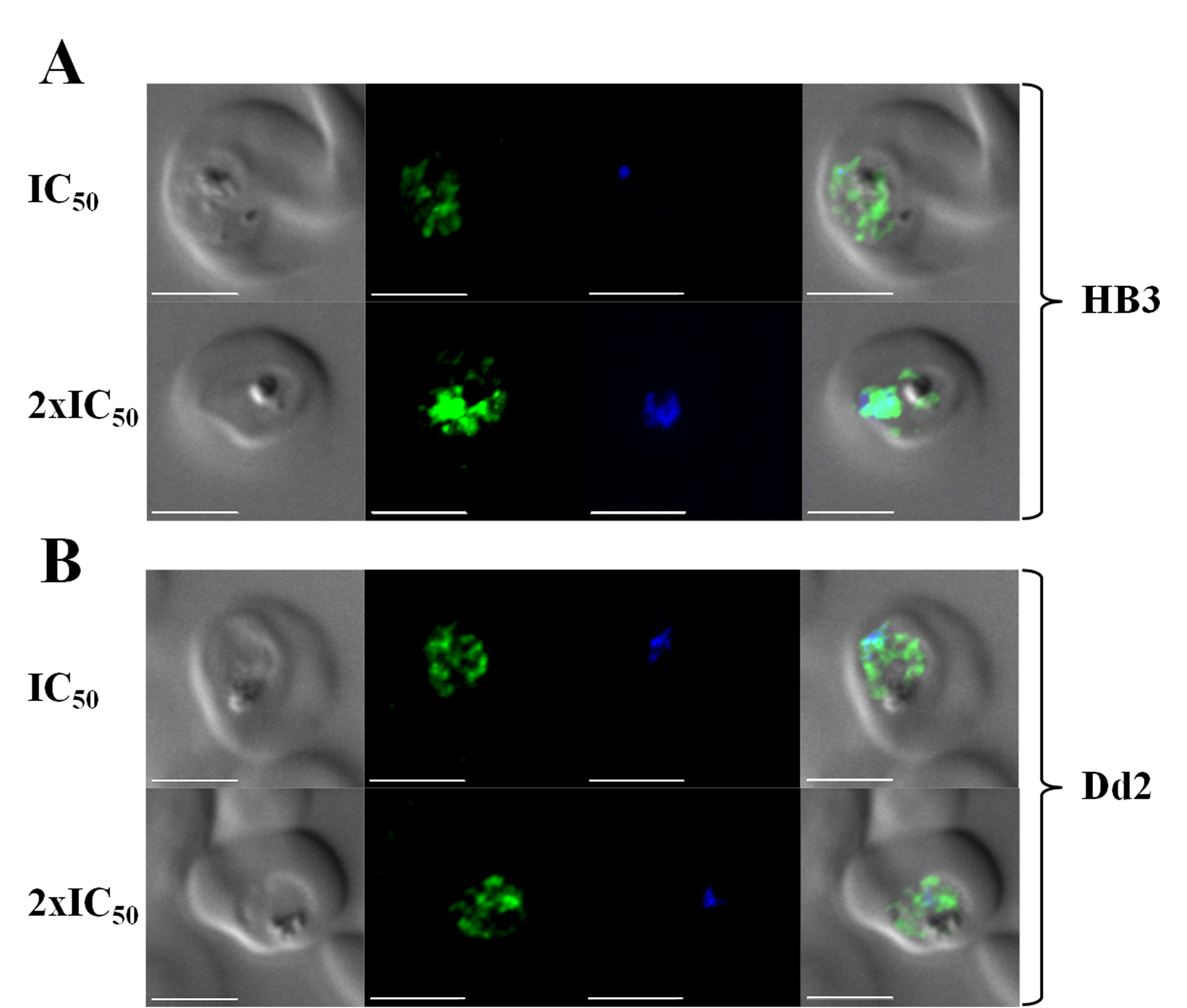

Supplement: Figure S2 — CQS (HB3, A) and CQR (Dd2, B) parasites treated with IC50 (top row each panel) and 2× IC50 (bottom row) doses of CQ (10, 20 nM and 125, 250 nM, respectively) for 48 hr. Parasites were then stained for ATG8 (green) and with DAPI (blue), as described in Methods. Very few peripherally disposed PfATG8 puncta are observed, supporting the conclusion that puncta formation is associated with LD50, but not IC50 doses of CQ. Scale bar = 5 µm. (DOC) [file pone.0079059.s002.doc]
