# Supplementary material for: A Process Similar to Autophagy Is Associated with Cytocidal Chloroquine Resistance in Plasmodium falciparum
Source: PLoS One. 2013 Nov 20;8(11):e79059. doi: 10.1371/journal.pone.0079059 (PMC3835802; doi:10.1371/journal.pone.0079059)
Supplement: Table S1 — LD50 values for the HB3×Dd2 cross progeny. (DOC) [file pone.0079059.s003.doc]

**Table S1. CQ LD_50_ values of the HB3 x Dd2 cross progeny.** Averages (+/- S.E.M.) from at least 3 independent experiments, each done in triplicate. Also shown is pfcrt allele expressed in the strains (“R” = Dd2, “S” = HB3 allele). See Methods and [6] for additional detail.

| ***Strain*** | ***pfcrt allele*** | ***CQ LD_50_ (nM)*** | ***S.E.M.*** |
| --- | --- | --- | --- |
| 7C170 | S | 36.5 | 2.2 |
| 7C188 | S | 41.0 | 2.9 |
| 7C159 | S | 41.2 | 3.7 |
| QC13 | S | 41.6 | 2.8 |
| B4R3 | S | 48.1 | 3.1 |
| CH3-61 | S | 48.4 | 4.0 |
| SC05 | S | 49.7 | 4.5 |
| QC01 | S | 50.7 | 4.3 |
| GC03 | S | 53.1 | 5.2 |
| 7C7 | S | 53.4 | 4.9 |
| 7C3 | S | 71.4 | 6.0 |
| TC08 | S | 78.8 | 7.3 |
| B1SD | S | 85.1 | 6.8 |
| 7C126 | S | 89.1 | 5.1 |
| 7C46 |  | 8047.5 | 654 |
| 7C183 | R | 11,747.2 | 40 |
| TC05 | R | 14,298.1 | 1621 |
| 7C424 | R | 15,129.5 | 877 |
| D43 | R | 18,251.2 | 1916 |
| QC34 | R | 18,455.7 | 1823 |
| 1BB5 | R | 19,798.1 | 1772 |
| 7C421 | R | 20,475.4 | 1303 |
| 7C12 | R | 20,478.0 | 1343 |
| 7C408 |  | 21,552.4 | 2169 |
| 3BD5 | R | 21,579.4 | 2107 |
| 7C111 | R | 23,778.1 | 1137 |
| SC01 | R | 23,997.2 | 2576 |
| GC06 | R | 27,394.9 | 1175 |
| 3BA6 | R | 31,837.2 | 2934 |
