# Supplementary material for: A Process Similar to Autophagy Is Associated with Cytocidal Chloroquine Resistance in Plasmodium falciparum
Source: PLoS One. 2013 Nov 20;8(11):e79059. doi: 10.1371/journal.pone.0079059 (PMC3835802; doi:10.1371/journal.pone.0079059)
Supplement: Table S2 — All genes within the chr6 LD50 locus. (DOC) [file pone.0079059.s004.doc]

**Table S2. All genes included in the LD50 chromosome 6 locus. HB3xDd2 CDS Score from PlasmoDB version 9.3 (2013) depicts the number of SNPs per 1000 basepairs.**

| **Gene ID PlasmoDB 9.3** | **Description** | **cM** | **HB3 x Dd2 CDS Score** | **Expression Correlation** |
| --- | --- | --- | --- | --- |
| PF3D7_0622100 | conserved Plasmodium protein, unknown function | 17.3 | 0.25 | - |
| PF3D7_0622200 | radical SAM protein, putative | 17.3 | 0.39 | - |
| PF3D7_0622300 | conserved Plasmodium protein, unknown function | 17.3 | - | 0.3 |
| PF3D7_0622400 | conserved Apicomplexan protein, unknown function | 17.3 | - | - |
| PF3D7_0622500 | RNA methyltransferase, putative | 17.3 | - | - |
| PF3D7_0622600 | conserved Plasmodium protein, unknown function | 17.3 | - | -0.22 |
| PF3D7_0622700 | conserved Plasmodium membrane protein, unknown function | 17.3 | - | 0.2 |
| PF3D7_0622800 | leucyl tRNA synthase | 17.3 | 0.69 | 0.15 |
| PF3D7_0622900 | transcription factor with AP2 domain(s), putative (ApiAP2) | 17.3 | 0.17 | 0.13 |
| PF3D7_0623000 | chorismate synthase (CS) | 17.3 | 1.26 | -0.33 |
| PF3D7_0623100 | coronin binding protein, putative | 17.3 | - | 0.15 |
| PF3D7_0623200 | ferredoxin NADP reductase (FNR) | 17.3 | 0.9 | -0.14 |
| PF3D7_0623300 | EGF-like membrane protein, putative | 17.3 | - | - |
| PF3D7_0623400 | RNA-binding protein mei2 homologue, putative | 17.3 | - | 0.11 |
| PF3D7_0623500 | superoxide dismutase (SOD2) | 17.3 | - | - |
| PF3D7_0623600 | transcription or splicing factor-like protein, putative | 17.3 | 0 | - |
| PF3D7_0623700 | ATP dependent DEAD-box helicase, putative | 17.3 | 0.59 | 0.43 |
| PF3D7_0623800 | protein kinase, putative (TKL4) | 17.3 | 1.11 | 0.19 |
| PF3D7_0623900 | ribonuclease HII, putative | 17.3 | 1.15 | -0.43 |
| PF3D7_0624000 | hexokinase (HK) | 17.3 | - | -0.24 |
| PF3D7_0624100 | conserved Plasmodium protein, unknown function | 14.4 | - | - |
| PF3D7_0624200 | conserved Plasmodium protein, unknown function | 14.4 | - | -0.24 |
| PF3D7_0624300 | conserved Plasmodium protein, unknown function | 14.4 | - | - |
| PF3D7_0624400 | conserved Plasmodium protein, unknown function | 14.4 | - | -0.43 |
| PF3D7_0624500 | anaphase promoting complex subunit, putative | 14.4 | - | -0.38 |
| PF3D7_0624600 | SNF2 helicase, putative (ISWI) | 11.5 | 0.12 | 0.01 |
| PF3D7_0624700 | N-acetylglucosaminylphosphatidylinositol deacetylase, putative | 11.5 | - | 0.09 |
| PF3D7_0624800 | conserved Plasmodium protein, unknown function | 11.5 | 0 | - |
| PF3D7_0624900 | conserved Plasmodium protein, unknown function | 11.5 | - | - |
| PF3D7_06250001 | phosphatidic acid phosphatase (PAP) | 2.9 | - | -0.09 |
| PF3D7_06250002 | phosphatidic acid phosphatase | 2.9 | - | -0.09 |
| PF3D7_0625100 | sphingomyelin synthase, putative | 2.9 | 0.83 | -0.11 |
| PF3D7_0625200 | conserved Plasmodium protein, unknown function | 2.9 | 0.86 | 0.1 |
| PF3D7_0625300 | DNA polymerase 1, putative | 2.9 | 0.46 | 0.12 |
| PF3D7_0625400 | conserved Plasmodium protein, unknown function | 2.9 | 1.61 | 0.39 |
| PF3D7_0625500 | conserved Plasmodium membrane protein, unknown function | 2.9 | - | 0.47 |
| PF3D7_0625600 | poly(A) polymerase PAP, putative | 2.9 | 0.53 | -0.21 |
| PF3D7_0625700 | conserved Plasmodium protein, unknown function | 2.9 | - | - |
| PF3D7_0625800 | conserved Plasmodium protein, unknown function | 2.9 | - | - |
| PF3D7_0625900 | conserved Plasmodium protein, unknown function | 2.9 | - | -0.38 |
| PF3D7_0626000 | conserved Plasmodium protein, unknown function | 2.9 | 0.38 | -0.08 |
| PF3D7_0626100 | oxidoreductase, short-chain dehydrogenase family, putative | 2.9 | 0.87 | 0.24 |
| PF3D7_0626200 | conserved Plasmodium protein, unknown function | 2.9 | - | -0.52 |
| PF3D7_0626300 | 3-oxoacyl-acyl-carrier protein synthase I/II (FabB/FabF) | 2.9 | - | 0.33 |
| PF3D7_0626400 | Sec14 domain containing protein | 2.9 | 2.59 | 0.12 |
| PF3D7_0626500 | conserved Plasmodium protein, unknown function | 2.9 | 1.05 | - |
| PF3D7_0626600 | conserved Plasmodium protein, unknown function | 2.9 | - | 0.05 |
| PF3D7_0626700 | conserved protein, unknown function | 2.9 | 0.89 | -0.16 |
| PF3D7_0626800 | pyruvate kinase (PyrK) | 2.9 | 1.3 | -0.32 |
| PF3D7_0626900 | mitochondrial ribosomal protein L46 precursor, putative | 2.9 | - | -0.38 |
| PF3D7_0627000 | conserved Plasmodium protein, unknown function | 2.9 | - | - |
| PF3D7_0627100 | ankyrin-repeat protein, putative | 2.9 | 0.19 | 0.21 |
| PF3D7_0627200 | myosin light chain, putative | 2.9 | 0 | 0.12 |
| PF3D7_0627300 | c3h4-type ring finger protein, putative | 2.9 | - | -0.17 |
| PF3D7_0627400 | mitochondrial import inner membrane translocase subunit, putative | 2.9 | - | -0.34 |
| PF3D7_0627500 | 4-methyl-5(B-hydroxyethyl)-thiazol monophosphate biosynthesis enzyme | 2.9 | - | -0.12 |
| PF3D7_0627600 | conserved Plasmodium protein, unknown function | 2.9 | 0.84 | 0.27 |
| PF3D7_0627700 | transportin | 2.9 | 1.16 | -0.14 |
| PF3D7_0627800 | acetyl-CoA synthetase, putative | 2.9 | 2.67 | -0.18 |
| PF3D7_0627900 | conserved Plasmodium protein, unknown function | 2.9 | - | 0.2 |
| PF3D7_0628000 | 6-pyruvoyltetrahydropterin synthase (PTPS) | 2.9 | - | -0.25 |
| PF3D7_0628100 | HECT-domain (ubiquitin-transferase), putative | 2.9 | 0.55 | 0.39 |
| PF3D7_0628200 | protein kinase PK4 (PK4) | 2.9 | 0.76 | 0.01 |
| PF3D7_0628300 | choline/ethanolaminephosphotransferase, putative (CEPT) | 0 | - | 0.24 |
| PF3D7_0628400 | conserved Plasmodium membrane protein, unknown function | 0 | - | 0.04 |
| PF3D7_0628500 | conserved Plasmodium protein, unknown function | 0 | 1.05 | - |
| PF3D7_0628600 | conserved Plasmodium protein, unknown function | 0 | - | 0.23 |
| PF3D7_0628700 | conserved Plasmodium protein, unknown function | 0 | 0.58 | -0.4 |
| PF3D7_0628800 | glutamyl-tRNA(Gln) amidotransferase subunit B, putative | 0 | 6.8 | -0.07 |
| PF3D7_0628900 | RAP protein, putative | 0 | 0 | -0.41 |
| PF3D7_0629000 | conserved protein, unknown function | 0 | - | - |
| PF3D7_0629100 | nicotinate phosphoribosyltransferase, putative | 0 | - | -0.32 |
| PF3D7_0629200 | DnaJ protein, putative | 0 | - | 0.03 |
| PF3D7_0629300 | phosphatidylcholine-sterol acyltransferase precursor, putative (PL) | 0 | 0.77 | 0.29 |
| PF3D7_0629400 | RNA binding protein, putative | 0 | - | -0.29 |
| PF3D7_0629500 | amino acid transporter, putative | 0 | 0.55 | -0.08 |
| PF3D7_0629600 | conserved Plasmodium protein, unknown function | 0 | - | - |
| PF3D7_0629700 | SET domain protein, putative (SET1) | 0 | 0.99 | -0.13 |
| PF3D7_0629800 | cullin-like protein, putative | 0 | 0.29 | - |
| PF3D7_0629900 | sec14-like cytosolic factor or phosphatidylinositol/ phosphatidylcholine transfer protein, putative | 0 | - | 0.39 |
| PF3D7_0630000 | CPW-WPC family protein | 0 | 0 | - |
| PF3D7_0630100 | conserved Plasmodium protein, unknown function | 0 | - | -0.03 |
| PF3D7_0630200 | secreted ookinete protein, putative (PSOP6) | 0 | - | - |
| PF3D7_0630300 | DNA polymerase epsilon, catalytic subunit a, putative | 0 | 0.69 | -0.21 |
| PF3D7_0630400 | conserved Plasmodium protein, unknown function | 0 | - | - |
| PF3D7_0630500 | microtubule-associated protein ytm1 homologue, putative | 0 | 1.34 | - |
| PF3D7_0630600 | conserved Plasmodium protein, unknown function | 0 | 3.91 | 0.07 |
| PF3D7_0630700 | conserved Plasmodium protein, unknown function | 0 | 0.51 | -0.39 |
| PF3D7_0630800 | conserved Plasmodium protein, unknown function | 0 | 3.38 | -0.05 |
| PF3D7_0630900 | DEAD/DEAH box ATP-dependent RNA helicase (Has1p) | 0 | 2.77 | 0.31 |
| PF3D7_0631000 | Tetratricopeptide repeat protein, putative | 0 | - | - |
| PF3D7_0631100 | Plasmodium exported protein (PHISTb), unknown function | 0 | - | - |
| PF3D7_0631200 | erythrocyte membrane protein 1 (PfEMP1), pseudogene  (VAR pseudogene) | 0 | - | - |
| PF3D7_0631300 | RESA-like protein | 0 | - | - |
| PF3D7_0631400 | Pfmc-2TM Maurer's cleft two transmembrane protein (MC-2TM) | 0 | - | -0.31 |
| PF3D7_0631500 | Plasmodium exported protein (hyp4), unknown function | 0 | - | 0.16 |
| PF3D7_0631600 | Plasmodium exported protein (hyp5), unknown function | 0 | - | 0.13 |
| PF3D7_0631700 | rifin, pseudogene | 0 | - | - |
| PF3D7_0631800 | rifin (RIF) | 0 | 9.72 | -0.24 |
| PF3D7_0631900 | stevor (stevor) | 0 | - | 0.03 |
| PF3D7_0632000 | rifin (RIF) | 0 | - | -0.09 |
| PF3D7_0632100 | rifin (RIF) | 0 | - | - |
| PF3D7_0632200 | rifin (RIF) | 0 | - | - |
| PF3D7_0632300 | rifin (RIF) | 0 | - | - |
| PF3D7_0632400 | rifin (RIF) | 0 | - | - |
| PF3D7_0632500 | erythrocyte membrane protein 1, PfEMP1 (VAR) | 0 | - | 0.05 |
| PF3D7_0632600 | rifin, pseudogene (RIF pseudogene) | 0 | - | - |
| PF3D7_0632700 | rifin (RIF) | 0 | - | - |
| PF3D7_0632800 | erythrocyte membrane protein 1, PfEMP1 (VAR) | 0 | - | -0.25 |
