# Supplementary material for: A Process Similar to Autophagy Is Associated with Cytocidal Chloroquine Resistance in Plasmodium falciparum
Source: PLoS One. 2013 Nov 20;8(11):e79059. doi: 10.1371/journal.pone.0079059 (PMC3835802; doi:10.1371/journal.pone.0079059)
Supplement: Table S3 — All genes within the LD50 chr6×chr8 interaction. (DOC) [file pone.0079059.s005.doc]

**Table S3. All genes included in the LD50 interaction Chr6 (cM11.5) x Chr8 (cM77.5). HB3xDd2 CDS Score from PlasmoDB version 9.3 (2013) depicts the number of SN**Ps per 1000 basepairs.

| **Gene ID**  **PlasmoDB 9.3** | **Chr** | **Description** | **HB3 x Dd2 CDS Score** | **Expression Correlation** |
| --- | --- | --- | --- | --- |
| PF3D7_0808000 | 8 | conserved Plasmodium protein, unknown function | 1.22 | -0.3 |
| PF3D7_0807900 | 8 | tyrosyl-tRNA synthetase, putative | - | -0.24 |
| PF3D7_0807700 | 8 | serine protease, putative | - | 0.4 |
| PF3D7_0807600 | 8 | conserved Plasmodium protein, unknown function | 4.24 | 0.13 |
| PF3D7_0807500 | 8 | proteasome subunit alpha, putative | - | -0.38 |
| PF3D7_0807300 | 8 | Rab GTPase 18 (RAB18) | - | 0.08 |
| PF3D7_0807200 | 8 | conserved Plasmodium membrane protein, unknown function | - | -0.07 |
| PF3D7_0807000 | 8 | gas41 homologue, putative | - | 0.41 |
| PF3D7_0806600 | 8 | kinesin-like protein, putative | 0.35 | 0.35 |
| PF3D7_0806400 | 8 | glycosyltransferase family 28 protein, putative | - | -0.01 |
| PF3D7_0806300 | 8 | ferlin like protein, putative | 0.59 | 0.38 |
| PF3D7_0806200 | 8 | conserved Plasmodium membrane protein, unknown function | 0.33 | -0.26 |
| PF3D7_0805900 | 8 | conserved Plasmodium protein, unknown function | 0 | 0.3 |
| PF3D7_0805800 | 8 | conserved Plasmodium protein, unknown function | 0 | 0.3 |
| PF3D7_0805100 | 8 | conserved Plasmodium protein, unknown function | - | 0.38 |
| PF3D7_0805000 | 8 | alpha/beta hydrolase, putative | - | -0.28 |
| PF3D7_0804500 | 8 | conserved Plasmodium membrane protein, unknown function | 1.58 | -0.36 |
| PF3D7_0804400 | 8 | methionine aminopeptidase 1c, putative (MetAP1c) | 0.45 | 0.21 |
| PF3D7_0804300 | 8 | conserved Plasmodium protein, unknown function | 2.99 | -0.01 |
| PF3D7_0803800 | 8 | 20S proteasome beta subunit | - | -0.03 |
| PF3D7_0805400 | 8 | acetyltransferase, putative | - | - |
| PF3D7_0805500 | 8 | conserved Plasmodium protein, unknown function | - | - |
| PF3D7_0805600 | 8 | apicoplast phosphatidic acid phosphatase, putative | - | - |
| PF3D7_0805700 | 8 | serine/threonine protein kinase, FIKK family (TSTK0) | - | - |
| PF3D7_0807400 | 8 | conserved Plasmodium protein, unknown function | - | - |
| PF3D7_0807800 | 8 | proteasome subunit alpha type 5, putative | - | -0.11 |
| PF3D7_0807100 | 8 | RNA helicase, putative | 0.25 | 0.09 |
| PF3D7_0806900 | 8 | conserved Plasmodium protein, unknown function | - | 0.01 |
| PF3D7_0806800 | 8 | vacuolar proton translocating ATPase subunit A, putative | - | 0.15 |
| PF3D7_0806700 | 8 | conserved Plasmodium membrane protein, unknown function | 1.94 | 0.13 |
| PF3D7_0806500 | 8 | DnaJ protein, putative | 0.49 | 0.18 |
| PF3D7_0806100 | 8 | conserved Plasmodium protein, unknown function | - | 0.18 |
| PF3D7_0806000 | 8 | AAA family ATPase, putative | 0.82 | 0.05 |
| PF3D7_0805300 | 8 | conserved Plasmodium protein, unknown function | 0.53 | 0.48 |
| PF3D7_0805200 | 8 | conserved Plasmodium protein, unknown function | - | 0.42 |
| PF3D7_0804900 | 8 | GTPase activator, putative | 0.68 | -0.39 |
| PF3D7_0804800 | 8 | peptidyl-prolyl cis-trans isomerase (CYP24) | - | -0.08 |
| PF3D7_0804700 | 8 | conserved Plasmodium protein, unknown function | 0.17 | 0.37 |
| PF3D7_0804600 | 8 | U2 snRNA/tRNA pseudouridine synthase, putative | - | -0.19 |
| PF3D7_0804000 | 8 | cactin homolog, putative | - | 0.12 |
| PF3D7_0804100 | 8 | small nucleolar RNA snoR06 | - | - |
| PF3D7_0803900 | 8 | unspecified product | - | - |
| PF3D7_0804200 | 8 | Plasmodium RNA of unkown function RUF3 | - | - |
| PF3D7_0624600 | 6 | SNF2 helicase, putative (ISWI) | 0.12 | 0.01 |
| PF3D7_0624700 | 6 | N-acetylglucosaminylphosphatidylinositol deacetylase, putative | - | 0.09 |
| PF3D7_0624800 | 6 | conserved Plasmodium protein, unknown function | 0 | - |
| PF3D7_0624900 | 6 | conserved Plasmodium protein, unknown function | - | - |
| PF3D7_0625000.1 | 6 | phosphatidic acid phosphatase (PAP) | - | -0.09 |
| PF3D7_0625000.2 | 6 | phosphatidic acid phosphatase | - | -0.09 |
| PF3D7_0625100 | 6 | sphingomyelin synthase, putative | 0.83 | -0.11 |
| PF3D7_0625200 | 6 | conserved Plasmodium protein, unknown function | 0.86 | 0.1 |
| PF3D7_0625300 | 6 | DNA polymerase 1, putative | 0.46 | 0.12 |
| PF3D7_0625400 | 6 | conserved Plasmodium protein, unknown function | 1.61 | 0.39 |
| PF3D7_0625500 | 6 | conserved Plasmodium membrane protein, unknown function | - | 0.47 |
| PF3D7_0625600 | 6 | poly(A) polymerase PAP, putative | 0.53 | -0.21 |
| PF3D7_0625700 | 6 | conserved Plasmodium protein, unknown function | - | - |
| PF3D7_0625800 | 6 | conserved Plasmodium protein, unknown function | - | - |
| PF3D7_0625900 | 6 | conserved Plasmodium protein, unknown function | - | -0.38 |
| PF3D7_0626000 | 6 | conserved Plasmodium protein, unknown function | 0.38 | -0.08 |
| PF3D7_0626100 | 6 | oxidoreductase, short-chain dehydrogenase family, putative | 0.87 | 0.24 |
| PF3D7_0626200 | 6 | conserved Plasmodium protein, unknown function | - | -0.52 |
| PF3D7_0626300 | 6 | 3-oxoacyl-acyl-carrier protein synthase I/II (FabB/FabF) | - | 0.33 |
| PF3D7_0626400 | 6 | Sec14 domain containing protein | 2.59 | 0.12 |
