# Supplementary material for: A Process Similar to Autophagy Is Associated with Cytocidal Chloroquine Resistance in Plasmodium falciparum
Source: PLoS One. 2013 Nov 20;8(11):e79059. doi: 10.1371/journal.pone.0079059 (PMC3835802; doi:10.1371/journal.pone.0079059)
Supplement: Table S4 — GO enriched molecular functions for the chr6 LD50 locus. (DOC) [file pone.0079059.s006.doc]

**Table S4. Enriched Molecular Functions for LD50 Chr 6 locus**

| **Term** | **Description** | **p-value** |
| --- | --- | --- |
| All transferase terms | transferase | 0.0024 |
| GO:0016740 | transferase activity | 0.0049 |
| GO:0003824 | catalytic activity | 0.0234 |
| GO:0016772 | transferase activity, transferring phosphorus-containing groups | 0.0357 |
| All zinc-finger terms | zinc-finger | 0.0380 |
| All nucleotidyltransferase terms | nucleotidyltransferase | 0.1294 |
