# Supplementary material for: A Process Similar to Autophagy Is Associated with Cytocidal Chloroquine Resistance in Plasmodium falciparum
Source: PLoS One. 2013 Nov 20;8(11):e79059. doi: 10.1371/journal.pone.0079059 (PMC3835802; doi:10.1371/journal.pone.0079059)
Supplement: Table S5 — GO enriched biological processes for the LD50 chr6 locus. (DOC) [file pone.0079059.s007.doc]

**Table S5. Enriched Biological Processes for LD50 Chr 6 locus**

| **Term** | **Description** | **p-value** |
| --- | --- | --- |
| GO:0016070 | RNA metabolic process | 0.0250 |
| All zinc-finger terms | zinc-finger | 0.0380 |
| GO:0034641 | cellular nitrogen compound metabolic process | 0.0821 |
| GO:0006807 | nitrogen compound metabolic process | 0.1114 |
| GO:0055114 | oxidation reduction | 0.2201 |
