# Supplementary material for: A Process Similar to Autophagy Is Associated with Cytocidal Chloroquine Resistance in Plasmodium falciparum
Source: PLoS One. 2013 Nov 20;8(11):e79059. doi: 10.1371/journal.pone.0079059 (PMC3835802; doi:10.1371/journal.pone.0079059)
Supplement: Table S6 — GO enriched molecular functions for the LD50 chr6×chr8 interaction. (DOC) [file pone.0079059.s008.doc]

**Table S6. Enriched Molecular Functions for LD50 Chr 6 x Chr 8 interaction locus**

| **Term** | **Description** | **p-value** |
| --- | --- | --- |
| All proteasome terms | proteasome | 0.0233 |
| GO:0070011 | peptidase activity, acting on L-amino acid peptides | 0.0586 |
| pfa03050 | Proteasome | 0.0712 |
| GO:0008233 | peptidase activity | 0.0817 |
| All Protease terms | Protease | 0.0870 |
| All hydrolase terms | hydrolase | 0.0902 |
| GO:0003824 | catalytic activity | 0.1578 |
| GO:0004175 | endopeptidase activity | 0.1623 |
