# Supplementary material for: A Process Similar to Autophagy Is Associated with Cytocidal Chloroquine Resistance in Plasmodium falciparum
Source: PLoS One. 2013 Nov 20;8(11):e79059. doi: 10.1371/journal.pone.0079059 (PMC3835802; doi:10.1371/journal.pone.0079059)
Supplement: Table S7 — GO enriched biological processes for the LD50 chr6×chr8 interaction. (DOC) [file pone.0079059.s009.doc]

**Table S7. Enriched Biological Processes for LD50 Chr 6 x Chr 8 interaction locus**

| **Term** | **Description** | **p-value** |
| --- | --- | --- |
| All proteasome terms | proteasome process | 0.0233 |
| pfa:03050 | proteasome process | 0.0712 |
| All Protease terms | Protease process | 0.0870 |
| All hydrolase terms | hydrolase process | 0.0902 |
| GO:0006508 | proteolysis | 0.0930 |
| GO:0019538 | protein metabolic process | 0.1329 |
