# Supplementary material for: A Process Similar to Autophagy Is Associated with Cytocidal Chloroquine Resistance in Plasmodium falciparum
Source: PLoS One. 2013 Nov 20;8(11):e79059. doi: 10.1371/journal.pone.0079059 (PMC3835802; doi:10.1371/journal.pone.0079059)

**Scheme S1:** Cartoon representation of 3D puncta quantification method used in the present work. Hemozoin density (red “x”) is used as the origin, and distances toPfATG8 positive puncta are defined for the reconstructed confocal z stack of images, relative to hemozoin, using 3D Cartesian (x,y,z) coordinates. The cartoon shows an abbreviated depiction of 3 SDCM “slices”, but as described in methods the z stack data set for each cell is a fully assembled, iteratively deconvolved 3D image constructed from approximately 15 – 20 z slices (see [35] for additional detail).


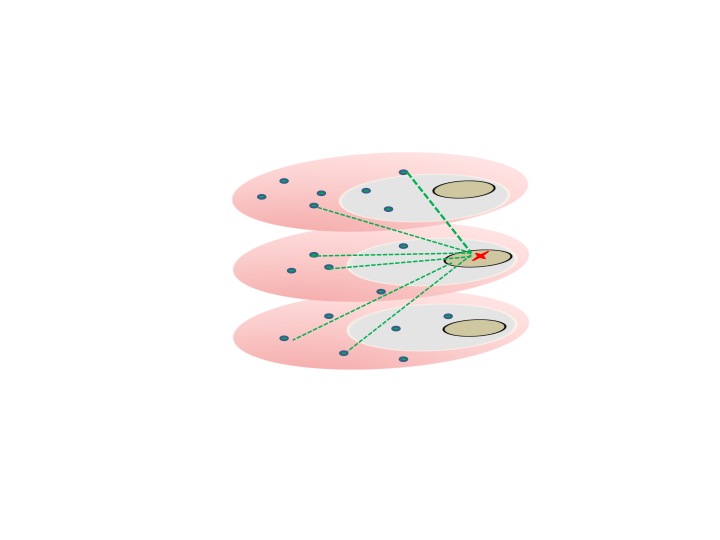

Supplement: Scheme S1 — Cartoon representation of 3D puncta quantification method used in the present work. Hemozoin density (red “x”) is used as the origin, and distances toPfATG8 positive puncta are defined for the reconstructed confocal z stack of images, relative to hemozoin, using 3D Cartesian (x,y,z) coordinates. The cartoon shows an abbreviated depiction of 3 SDCM “slices”, but as described in methods the z stack data set for each cell is a fully assembled, iteratively deconvolved 3D image constructed from approximately 15–20 z slices (see [35] for additional detail). (DOC) [file pone.0079059.s010.doc]
